# Single-cell and spatial transcriptomic analysis reveal cellular heterogeneity and cancer cell-intrinsic major histocompatibility complex II expression in urothelial carcinoma

**DOI:** 10.7150/ijbs.114618

**Published:** 2025-10-20

**Authors:** Shengwei Xiong, Jian Fan, Cong Huang, Shiming He, Yuan Liang, Qi Zhang, Bin Guo, Yucai Wu, Mancheng Xia, Fangzhou Zhao, Weimin Ci, Liqun Zhou, Yanqing Gong, Xuesong Li

**Affiliations:** 1 Department of Urology, Peking University First Hospital, Beijing, 100034, China.; 2 Institution of Urology, Peking University, Beijing, 100034, China.; 3 Beijing Key Laboratory of Urogenital Diseases (Male) Molecular Diagnosis and Treatment Center, Beijing, 100034, China.; 4 National Urological Cancer Center, Beijing, 100034, China.; 5 Department of Urology, Chinese PLA General Hospital, Beijing 100039, China.; 6 China National Center for Bioinformation, Beijing Institute of Genomics, Chinese Academy of Sciences, Beijing, 100101, China.

**Keywords:** Urothelial carcinoma, Single-cell RNA sequencing, Cellular heterogeneity, MHC-II molecules, Muscle invasion, Immunosuppressive microenvironment

## Abstract

Muscle-invasive (MI) urothelial carcinoma (UC) is a clinically challenging malignancy with a poor prognosis. Understanding the cellular dynamics that drive UC progression is critical for the development of optimized therapeutic strategies. Through integrative analysis of large-scale single-cell transcriptomic datasets from non-muscle-invasive (NMI) and MI tumours and validation with spatial transcriptomic datasets, we systematically characterized immune cell dynamics and cancer cell plasticity during UC progression. Our analysis revealed an immunosuppressive tumour microenvironment and a subset of cancer cells with upregulated major histocompatibility complex II (MHC-II) expression in MI tumours. Notably, MHC-II⁺ cancer cells were induced by interferon-γ signalling, as confirmed through *in vitro* experiments, and exhibited phenotypic alterations characterized by enhanced proliferative and migratory capacities. Furthermore, MHC-II⁺ cancer cells spatially colocalized with CD8⁺ T cells, regulatory T cells, and SPP1⁺ macrophages, where they engaged with inhibitory receptors on these immune cells, promoted CD8⁺ T cell exhaustion and facilitated immune evasion.

## Introduction

Urothelial carcinoma (UC), a malignancy originating in the urothelium, is one of the most prevalent genitourinary cancers 1. Approximately 90-95% of UC cases arise in the bladder, while the remaining 5%-10% occur in the upper urinary tract (renal pelvis and ureter) 2. Although urothelial carcinoma of the bladder (UCB) and upper tract urothelial carcinoma (UTUC) share histologic similarities and common risk factors, such as smoking, phenacetin use, and occupational exposure 3, 4, omics analyses have revealed distinct molecular profiles 5. For example, UTUC is characterized predominantly by a luminal papillary phenotype and exhibits a T-cell-depleted immune microenvironment 6, 7. Furthermore, patients who progress to muscle-invasive (MI) UC have significantly worse survival outcomes than those with non-muscle-invasive (NMI) tumours, a pattern consistent with that of both UCB and UTUC 8. Therefore, clarification of the mechanisms underlying UC progression to MI cancer is critical. Current biomarkers of tumor progression are associated with cell cycle regulation, MAPK signalling, apoptosis, chromatin stability, and the DNA damage response 9. However, most of these biomarkers were identified through omics-based risk stratification or molecular subtyping, which limits insights into the specific contributions of certain cell types to tumour progression.

Single-cell RNA sequencing (scRNA-seq) has emerged as a powerful tool for elucidating cellular heterogeneity, differentiation trajectories, and intercellular communication. Recent studies leveraging this technology have provided critical insights into intratumoral heterogeneity within UC, and have identified key cell types implicated in tumorigenesis and progression, including cytotoxic CD4^+^ T cell subsets, invasive cancer cell subpopulations, and cancer-associated fibroblasts 10-15. Nevertheless, systematic comparisons of UC at single-cell resolution remain limited, particularly in the following contexts: (1) direct molecular contrasts between UCB and UTUC; (2) differences between NMI and MI UC; and (3) variations across distinct molecular subtypes of UC.

In this study, we conducted an integrated analysis combining in-house and publicly available scRNA-seq datasets to systematically characterize cellular heterogeneity within the UC tumour microenvironment, with a specific focus on delineating both shared and distinct molecular features between UCB and UTUC at single-cell resolution. Furthermore, we performed a comprehensive investigation into the cellular and molecular mechanisms that drive UC progression to MI cancer, which was supported by spatial transcriptomic validation.

## Methods and Materials

### Sample collection and online dataset acquisition

Four UTUC samples were obtained from four patients who underwent laparoscopic radical nephroureterectomy (RNU) at Peking University First Hospital. Pathological analysis confirmed that two patients had NMI ureteral UC, while the remaining patients had MI UC of the renal pelvis and ureter, respectively. These four UTUC samples were subjected to spatial transcriptomics sequencing. In addition, two normal ureteral samples were collected from two patients who underwent ureteral reconstruction at the same institution and were submitted for scRNA-seq. Samples were collected after written informed consent was obtained from all patients. The study was approved by the Biomedical Research Ethics Committee of Peking University First Hospital (No. 2022[Yan200]).

**Figure 1:**
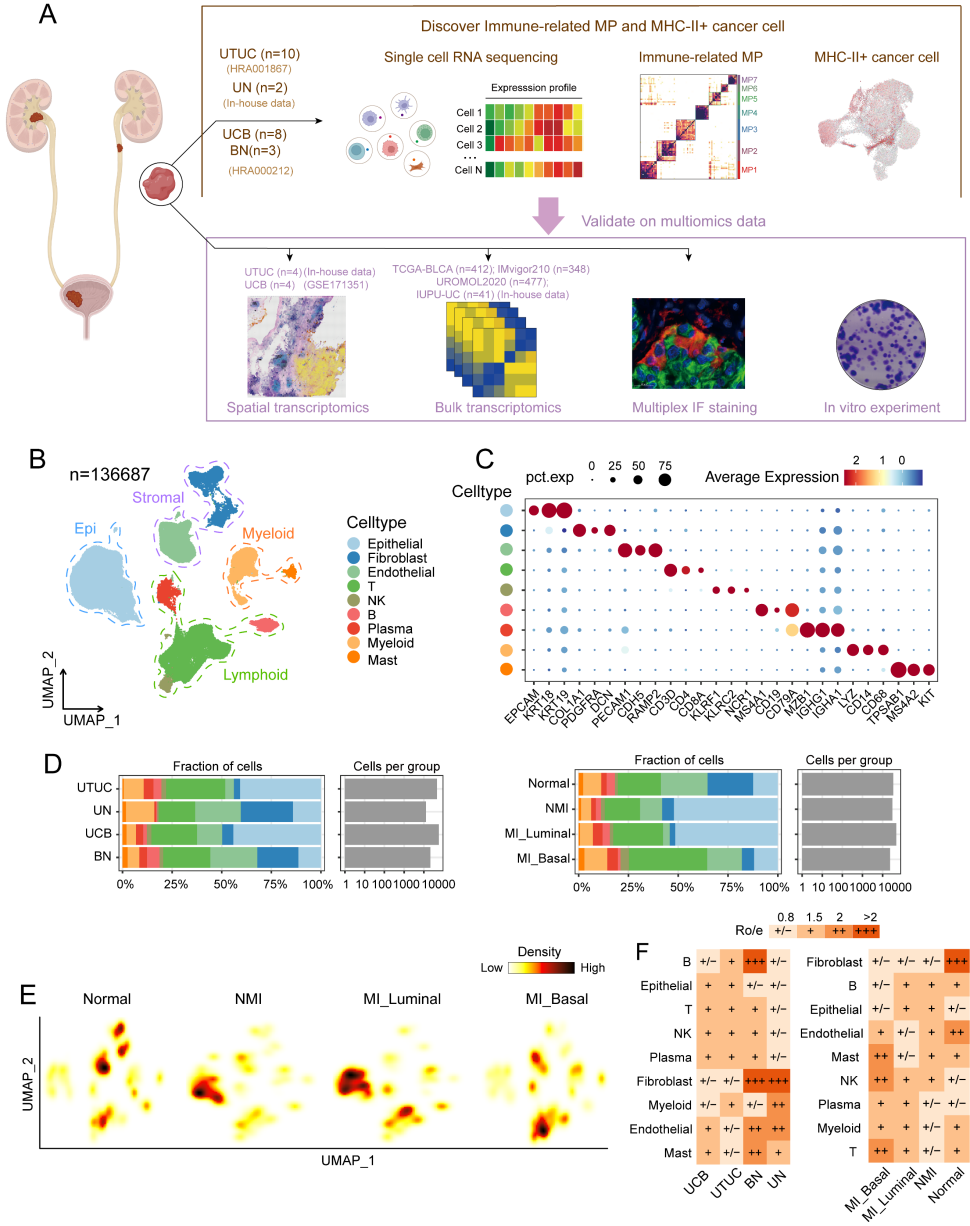
Single-cell transcriptomics reveal the cellular heterogeneity of UC.** (**A) Schematic illustration of sample collection and analysis workflow. (B) UMAP plot of 136,687 single cells, colored by nine major cell types. (C) Violin plot showing representative marker genes for the major cell types. (D) Cell fraction (as percentages) and cell number per group, stratified by molecular subtype (left) and tissue type (right). (E) Cell density plots stratified by molecular subtype. (F) Distribution of the ratio of observed to expected cell numbers (R_o/e_) for each cell type across tissue type (left) and molecular subtype (right), as estimated by the STARTRAC-dist index. A R_o/e_ value > 1 indicates enrichment of a given cell type, whereas a R_o/e_ value < 1 indicates depletion. Abbreviations: UTUC, upper tract urothelial carcinoma; UN, normal ureteral mucosa; UCB, urothelial carcinomas of the bladder; BN, normal bladder mucosa; MP, meta-program; IF, immunofluorescence.

ScRNA-seq data of 18 tumor tissues (Table S1), including eight UCB and ten UTUC cases, as well as three samples of adjacent normal bladder tissues, were obtained from Chen et al. 12 and Liang et al. 13 under the accession code HRA000212 and HRA001867, respectively, in the Genome Sequence Archive (GSA) for Human. Spatial transcriptomics data of four UCB tissues (Table S2) were retrieved from Gouin et al 16 under the accession number GSE171351 in the Gene Expression Omnibus (GEO). Additionally, bulk RNA-seq data for NMI UCB (n=477) were downloaded from the European Genome-Phenome Archive (EGA) (accession ID EGAD00001006656) 17. Bulk RNA-seq data for muscle-invasive UCB (n = 431) were obtained from The Cancer Genome Atlas (TCGA) database. Bulk RNA-seq data for metastatic UC (n=348) were retrieved from the IMvigor210 cohort. In addition, we generated an internal transcriptome sequencing cohort, IUPU-UC, comprising 41 samples (Figure 1A), the detailed information of which is provided in Table S3.

### Single-cell suspension preparation and scRNA-seq

The fresh ureteral mucosal tissues of two patients were stored in the MACS^®^ Tissue Preservation Solution (Miltenyi Biotec, Germany) on ice within 30 minutes after surgery. After they were transported to the laboratory, single-cell suspensions were prepared through a combination of mechanical and enzymatic dissociation. The cell suspension was subsequently processed using the Single-Cell 3' Library and Gel Bead Kit V3.1 (10x Genomics, 1000075) along with the Chromium Single Cell B Chip Kit (10x Genomics, 1000074). In accordance with the manufacturer's instructions, the suspension was loaded onto a Chromium single-cell controller (10x Genomics) to generate the cDNA library. Finally, cDNA libraries were prepared and sequenced on an Illumina NovaSeq 6000 platform using a paired-end 150 bp (PE150) reading strategy.

### scRNA-seq data processing, integration and clustering

Standard pipelines of the Cell Ranger Single-Cell toolkit (v7.0.0, 10X Genomics) were used to process FASTQ files of both in-house and online public samples to produce gene expression matrices based on the human reference genome (GRCh38-2020-A). The Seurat R package (v4.4.0) was used to subsequently analyse the scRNA-seq data. Low-quality cells with fewer than 200 or more than 8000 expressed genes, and cells with more than 10% mitochondrial RNA content were filtered out and removed. The DoubletFinder 18 R package (v2.0.3) was used to remove potential doublets, with an assumed doublet rate of 0.075. The SCTransform method was applied to normalize and stabilize the variances of the scRNA-seq datasets for each individual sample, and regression accounted for the mitochondrial and ribosomal contents. All individual samples were integrated by using the reciprocal principal component analysis (PCA) pipeline to eliminate batch effects. We subsequently identified the top 2,000 highly variable genes for PCA, after which the top 40 principal components were selected for uniform manifold approximation and projection (UMAP) dimension reduction. Clustering was performed using shared nearest-neighbour graph construction (FindNeighbors) followed by the FindClusters function, which allowed a range of resolutions between 0.5 and 2. Analysis of differentially expressed genes (DEGs) was conducted across clusters generated at a resolution of 1 using the Wilcoxon rank sum test and the “FindAllMarkers” function. The major cell types were assigned to each cluster were related to the expression patterns of the canonical marker genes. For each sample, the proportion of each cell type was calculated by dividing the number of cells of that type by the total number of cells and multiplying by 100 to obtain a percentage.

### Malignant cell identification based on inferred CNV

Malignant cells were identified among epithelial cells in tumour samples using the inferCNVpy algorithm (version 0.4.2; https://github.com/icbi-lab/infercnvpy). The function infercnvpy.tl.infercnv was applied to immune cells as normal references, to infer copy number variation (CNV). Subsequent dimensionality reduction was performed using PCA, followed by clustering on the basis of CNV profiles. The function infercnvpy.tl.umap was used to visualize CNV patterns. The CNV scores for each cell were calculated using the cnv.tl.cnv_score function. Cells were deemed malignant if they met the following criteria: (i) they demonstrated the ability to form separate clusters, and (ii) they had higher CNV scores than those of reference cells or known normal cell types.

### Defining meta-programs of malignant cells using cNMF

To capture tumour heterogeneity, we employed nonnegative matrix factorization (NMF) in the malignant cells of each sample. NMF was performed for each sample using the consensus NMF (cNMF) (v.1.3) (https://github.com/dylkot/cNMF). This algorithm decomposes a single-cell count matrix (N cells × G genes) into two nonnegative matrices: a gene expression program matrix (K × G) and a program usage matrix (N × K), which elucidate the contribution of individual genes to each program and the cell-specific patterns of program usage. The parameter K represents the number of gene expression programs to be inferred. First, we performed data preprocessing to filter out mitochondrial and ribosomal genes. Subsequently, cNMF was performed with K values ranging from 4 to 12, using 200 independent replicates for each value to ensure robust results, thereby generating 72 programs per sample. Programs that recurred across different K values and samples were defined as robust NMF programs according to the method outlined by Gavish et al. 19. Ultimately, a total of 133 robust NMF programs were identified.

We next clustered the robust NMF programs to identify the meta-programs (MPs) on the basis of similarity (Jaccard index), which was calculated by the iteratively selection of robust programs according to gene overlap 19. Each MP consists of the top 30 genes with the greatest overlap across programs. The final MPs were obtained after those that contained programs from only a single sample were excluded. We subsequently annotated the MPs by assessing their enrichment in functional gene sets, primarily using gene sets from MsigDB, Hallmark, and CancerSEA 20.

### Cell type distribution analysis

To assess the distribution of cell clusters across different phenotypic states, we quantified the relative abundance of each cluster within the total cell population or among major cell types in samples categorized by pathological conditions. To determine the degree of enrichment or depletion of specific cell clusters in relation to the phenotypic context, we computed the **r**atio of **o**bserved to **e**xpected cell counts (R_o/e_) for each tissue type and phenotype. The expected cell numbers for each cell cluster were obtained using the STARTRAC-dist index through the χ^2^ test 21. A Ro/e value greater than 1 was indicated as enrichment of a cell subset within a particular phenotype, whereas a value less than 1 indicated depletion.

### Cell subcluster identification

The major cell lineages, including T cells, NK cells, myeloid cells, fibroblasts, and endothelial cells, were then isolated for subsequent reclustering analysis. For each cell lineage, the harmony algorithm (v0.1.0) was employed to mitigate potential batch effects. Following batch effect correction, dimensionality reduction, graph-based clustering, and UMAP visualization were performed. DEGs for each subcluster were identified using the “FindAllMarkers” function in Seurat with default parameters. Cell subclusters were subsequently annotated according to the the expression profiles of DEGs and canonical marker genes. T cells and NK cells were reclustered and annotated on the basis of function-associated signatures according to previous studies 13, 22, and were further validated using the TCellSI 23 method. Macrophage polarization was assessed by calculating M1 and M2 polarization gene signature scores (Table S4), as defined by Liu et al. 24. The characterization of endothelial cell phenotypes was performed using gene signatures (Table S4) specific to tip and stalk cells 25. Per-cell scores were calculated using Seurat's AddModuleScore function, which computes the average expression of gene signatures relative to control genes, enabling quantification across cell subclusters.

### Transcriptomic molecular subtypes of MI UC

The pathological classification of NMI and MI is based on the diagnosis provided by the pathologists. Bulk RNA-seq samples from patients with MI UC were further classified into luminal and basal phenotypes according to the BASE47 gene set (**b**ladder cancer **a**nalysis of **s**ubtypes by gene **e**xpression) (Table S4) 26, and this classification was subsequently validated using the BLCAsubtyping software (v2.1.1; available at https://github.com/cit-bioinfo/BLCAsubtyping). We retrieved the gene expression matrix for thirty-seven bladder cancer cell lines from the Cancer Cell Line Encyclopedia (CCLE) database (https://sites.broadinstitute.org/ccle/). These cell lines were subsequently classified into luminal and basal phenotypes using the same method applied to the bulk RNA-seq data. For scRNA-seq and spatial transcriptomics data from patients with MI UC, we generated pseudobulk data by aggregating expression profiles within each sample, followed by categorization into luminal and basal subtypes using the same classification approach. Furthermore, we calculated basal and luminal signature scores for all the scRNA-seq samples using the BASE47 gene set and the AUCell algorithm, which ranks gene expression per cell and computes an AUC score for each gene set. Per-sample scores were obtained by averaging the AUC scores across all cells in each sample and were used for subsequent correlation analyses.

### Cell type deconvolution in bulk RNA-seq data

The BayesPrism algorithm 27 was used to investigate the cell type abundance in the bulk RNA-Seq data. In comparison to CIBERSORTx, BayesPrism integrates both a deconvolution module and an embedding learning module that employs the expectation maximization technique to accurately estimate tumour composition by linearly combining malignant gene programs. This approach enhances the precision of tumour microenvironment characterization 27. Annotated immune cell subclusters and cancer cell subsets served as reference cell type-specific expression profiles for the deconvolution of bulk RNA-seq data from the TCGA-BLCA cohort and the UROMOL 2020 4 cohort. The cell type abundances were subsequently compared across tissue subtypes and molecular subgroups, after which a correlation analysis were performed to explore the relationships among the identified cell subclusters.

### Pseudotime analysis

We used the R package monocle3 (v1.3.1) (https://cole-trapnell-lab.github.io/monocle3/) to perform pseudotime analysis, and aimed to investigate the differentiation trajectories of T-cell and macrophage subsets. To define the initial point of the pseudotime trajectory, we utilized CytoTRACE 28 to estimate the differentiation potential of individual cells. Cell subsets with higher CytoTRACE scores, which are indicative of a lower degree of differentiation, were considered as the root cells for the pseudotime analysis. Subsequently, we visualized both cell density and gene expression patterns along the pseudotime axis using the R packages *ggridges* and *ClusterGVis* (https://github.com/junjunlab/ClusterGVis).

### Transcription factor analysis

To evaluate transcription factor activity within cancer cell subsets, we performed single-cell regulatory network inference and clustering (SCENIC) analysis using the pySCENIC package (v0.12.1) 29 in Python (v3.9) with default parameters. The coexpression network was constructed using GRNboost2, and regulons were identified through RcisTarget. The activity of each regulon in individual cells was quantified using the AUCell algorithm, which provides scores that reflect the transcriptional activity across the cellular landscape.

### Cell-cell communication analysis

We utilized CellPhoneDB (v5.0) (https://github.com/ventolab/CellphoneDB) to infer potential ligand-receptor interactions between cancer cell subsets and immune cell subsets within the tumour microenvironment. This updated toolkit leverages a comprehensive, manually curated database of receptors, ligands, and their interactions, thereby improving the precision of cell-cell communication analysis. To compare cell-cell interactions between the cellular components of the MI and NMI groups, we adopted the MultiNicheNet cell-cell communication analysis framework (v2.0.0) (https://github.com/saeyslab/multinichenetr), which is specifically designed for multi-sample, multi-condition scRNA-seq datasets. Ligand-receptor pairs exhibiting statistically significant interactions (*P* value < 0.05) were identified and extracted for further visualization.

### Survival analysis

The bulk RNA-seq data from the TCGA-BLCA, IMvigor210 and in-house IUPU-UC cohort were used for survival analysis. Cell type-specific gene signatures were defined on basis of the top 50 marker genes identified using the COSine similarity-based marker Gene identification (COSG) method 30. The signature score for each cell type was computed, and patients were stratified into high- and low-signature score groups according to an optimal cut-off value determined by the *survminer* R package (v0.4.9). Kaplan-Meier (KM) survival curves were subsequently generated using the *survival* R package (v3.2.11) to assess the prognostic significance of the identified cell type signatures.

### Tissue preparation and data preprocessing for spatial transcriptomics

Tissue sections from four UTUC samples were processed according to the tissue preparation guide of Visium Spatial Gene Expression tissue preparation guide for fresh-frozen tissues (10x Genomics, CG000636). The tissue sections were mounted onto Visium Spatial Tissue Optimization Slide and subjected to methanol fixation and hematoxylin‒eosin staining. The optimal permeabilization time was determined and applied to facilitate efficient transcript capture. Barcoded cDNA synthesis, amplification, and library construction were conducted using Visium Spatial Gene Expression Reagent Kits (10x Genomics). Finally, sequencing was conducted on an Illumina NovaSeq 6000 platform in 150 bp paired-end mode.

Visium raw sequencing data were processed using Space Ranger (v1.3.0, 10x Genomics) to generate gene expression count matrices. Reads were aligned to the hg38 (GRCh38-2020-A) human reference genome and mapped against the corresponding probe set reference for humans. After preprocessing, spots with fewer than 500 counts, or fewer than 300 measured genes were removed. Additionally, mitochondrial genes were filtrated for downstream analysis. SCTransform-based normalization, dimensional reduction and clustering analysis were conducted using the Seurat R package (v4.4.0) under default parameters.

### Deconvolution and colocalization analysis of Visium data

To infer the cellular composition of each Visium spot, we employed cell2location (v0.1.3) 31 for cell type deconvolution. Prior to deconvolution, a permissive gene selection was performed using default parameters to define reference cell type signatures from the scRNA-seq data, and model training was configured for 500 epochs. The cell2location model was subsequently trained for 30,000 epochs, and the 5% quantile of the estimated posterior distribution of cell abundance was extracted and stored in an AnnData object. To delineate cellular niches across tissue sections, we applied the NMF function of cell2location to identify distinct cellular compartments. Colocalization analysis was conducted using the run_colocation function with default settings, and the optimal number of factors was determined manually the basis of biological relevance. We used mistyR (version 1.8.0) 32 to determine the importance of the intrinsic view within a spot by modelling cell type cell2location estimations of the NMI and MI phenotype slides. Pairwise Kullback-Leibler (KL) divergences between cell types were calculated using a symmetric KL function 33. To assess the statistical significance of spatial colocalization, a null distribution of KL values was generated by randomly sampling 80% of the spots from each cell type and computing the KL divergence over 1000 permutations. Empirical p-values were calculated as the fraction of null KL values exceeding the observed KL divergence. The differences in KL values compared with the observed values among cell types are illustrated as heatmaps. In addition, the observed KL values were plotted against the null distribution using density plots, with cell types coloured for clarity.

### Inference of pathway activity using PROGENy

To infer signalling pathway activities in cancer cell subsets, we applied the PROGENy (v1.28.0) R package 34 with default parameters. The pathway enrichment scores were averaged and analysed across pathological and molecular subgroups to identify pathway-specific differences. For the Visium data, we used the multivariate linear model (mlm) method implemented in the decoupleR package (v1.9.2) 35 to estimate pathway enrichment scores. Specifically, for each Visium spot, pathway activity was inferred according to the top 500 genes responsive to the PROGENy model, ranked by statistical significance (p value).

### Multiplex immunofluorescence staining

Formalin-fixed, paraffin-embedded (FFPE) tissue sections from UC patients were deparaffinized, rehydrated, and rinsed with distilled water. Antigen retrieval was performed using citrate buffer. Multiplex immunofluorescence staining was conducted with a fluorescent immunohistochemistry kit (Beyotime), according to the manufacturer's protocol. Primary antibodies targeting pan-CK, HLA-DRA, and CD68 (Proteintech) were applied to the sections, which were subsequently incubated overnight at 4 °C. The slides were then treated with HRP-conjugated secondary antibodies (mouse & rabbit) for 10 minutes at room temperature, followed by tyramide signal amplification. After completing antibody staining was complete, the cell nuclei were counterstained with DAPI. The slides were scanned using a Zeiss LSM900 confocal microscope, after which the images were further processed using SlideViewer (v2.8) software.

### Cell lines culture and treatment

The human bladder cancer cell lines T24 and 5637 were obtained from the American Type Culture Collection (ATCC, Manassas, VA, USA). Both cell lines were cultivated in RPMI-1640 medium supplemented with 10% foetal bovine serum (FBS) and 1% penicillin‒streptomycin. Cultures were incubated in a humidified atmosphere containing 5% CO₂ at 37 °C. For experimental treatments, T24 and 5637 cells were exposed to recombinant human IFN-γ (MedChemExpress) at concentrations of 0, 5, 10, and 20 ng/mL for 72 hours and maintained at 37 °C. To completely block the JAK signalling pathway, cells were pretreated with the selective JAK1/2 inhibitor ruxolitinib (MedChemExpress, 10 and 20 μM) for 1 hour prior to IFN-γ stimulation and were exposed to the inhibitor throughout the 72-hour treatment period.

### Lentiviral transduction and screening for stable cell lines

The HLA-DRA-overexpression (HLA-DRA-OE) and control lentiviral vectors were constructed using a Lentiviral Packaging Kit (Yeasen). Lentiviral transduction was performed according to the manufacturer's instructions to generate HLA-DRA-OE and control (HLA-DRA-NC) cell lines. Cells with stable expression were selected by culturing in medium supplemented with 10 μg/mL blasticidin (Yeasen) for two weeks.

### Western blot analysis

Cells were cultured in 10-cm plates and lysed in RIPA complete lysis buffer (Beyotime) for total protein extraction. After quantification and denaturation, 20 μg of proteins were separated by 8-12% SDS-PAGE and transferred onto a PVDF membrane (Millipore). The membrane was blocked with 5% bull serum albumin and incubated with primary antibodies (Cell Signaling Technology) (Jak1, 1:1000; phospho-Jak1(Tyr1034/1035), 1:1000; Stat1, 1:1000; phospho-Stat1 (Tyr701), 1:1000; HLA-DRA, 1:10000, β-tubulin, 1:5000) overnight at 4 °C, followed by by incubation with secondary antibodies. Protein signals were detected using the BeyoECL Plus chemiluminescent reagent (Beyotime).

### Proliferation and migration assay

Cell Counting Kit-8 (CCK-8, Beyotime) and colony formation assays were used to assess cell proliferation. HLA-DRA-OE and control T24 and 5637 cells (1,000/well) were seeded in 96-well plates and cultured. Cell viability was measured at 0, 1, 2, 3, 4, and 5 days by adding 10 μL CCK-8 solution to 90 μL medium, followed by incubation at 37 °C for 2 h and absorbance detection at 450 nm using a microplate reader (Bio-Rad). For colony formation, 1,000 cells were seeded into six-well plates and cultured until visible colonies formed. Colonies were fixed in 4% paraformaldehyde (PFA) for 15 min and stained with 0.1% crystal violet for 15 min. Colonies containing > 50 cells were counted for analysis.

Wound-healing assay was applied to evaluate cell migration. Cells were seeded in six-well plates and grown to 80-90% confluence. A linear scratch was made using a sterile pipette tip, and cells were cultured in medium containing 2% FBS for 24 h. Wound closure was imaged at 0 and 24 h with a digital microscope, and migration distance was quantified using ImageJ.

Transwell assays were further used to assess cell migration and invasion. A total of 2 × 10⁴ T24 cells or 5 × 10⁴ 5637 cells in 100 μL serum-free medium were seeded into the upper chambers (8 μm pore size, Corning) precoated with Matrigel (1:8 dilution, 50 μL/well) for invasion assays or left uncoated for migration assays. The lower chambers contained 500 μL medium containing 10% FBS. After incubation at 37 °C for 24-48 h, the migrated or invaded cells were fixed in 4% PFA and stained with 0.1% crystal violet before visualization and quantification.

### Statistical analysis

All the statistical analyses and data visualizations were performed using R (v4.3.2). Differences between two independent groups were assessed using the Wilcoxon rank-sum test for nonparametric data and Student's t test for normally distributed data. Comparisons among multiple groups were conducted using one-way ANOVA, followed by post hoc tests where appropriate. All p-values were two-sided, and p < 0.05 considered indicated statistical significance.

## Results

### Single-cell transcriptomic atlas of UC and normal samples

To determine the heterogeneity within UC and its potential mechanisms of progression, we analysed the single-cell transcriptomic profiles of ten UTUC samples, two normal ureter tissue samples, eight UCB tissue samples, and three adjacent bladder mucosa tissue samples (Figure 1A and Table S1). We classified the MI UC samples into luminal and basal phenotypes according to the BASE47 gene sets 26 (Figure S1A and S1B, Table S4). After stringent quality control and dimensionality reduction, we obtained transcriptional profiles of a total of 136,687 cells, which were broadly categorized into nine major cell types on basis of the expression of canonical marker genes; cell types included epithelial cells, fibroblasts, endothelial cells, T cells, NK cells, B cells, plasma cells, myeloid cells and mast cells (Figure 1B and 1C). These cell types were common between the pathological and molecular subtypes, although their proportions varied across these subtypes (Figure 1D). Stromal cells, including fibroblasts and endothelial cells, were significantly depleted in both NMI and MI tumour samples (Fig. 1E and 1F). The abundance of tumour-infiltrating T cells was significantly greater in the MI samples than in the NMI samples, with the most pronounced increase observed in the MI basal subgroup. These findings suggest a potential association between MI progression and enhanced T-cell infiltration. Additionally, the proportions of epithelial cells were elevated in tumour samples compared with normal tissues but were notably reduced in the MI basal subgroup (Figure 1D, 1E and 1F).

We further explored single-cell transcriptomic differences within the UTUC and UCB ecosystems. Our analysis revealed a similar distribution of major cell types across both tumour types (Figure 1F, S1C), although the proportions of certain cell types varied. Specifically, mast cells and endothelial cells were more abundant in UCB, whereas myeloid and B cells were enriched in UTUC (Figure S1D). DEG analysis of major cell types revealed that many genes, including mitochondrial genes and those that encode noncoding RNAs, were significantly differentially expressed between UCB and UTUC (Figure S1D). Hallmark pathway enrichment analysis, which focused on cell types with notable differences, revealed several tumour-associated pathways, including oxidative phosphorylation and TGF-beta signalling in mast cells (Figure S1F), protein secretion in endothelial cells (Figure S1G), and angiogenesis in B cells (Figure S1H). Whether these pathways represent true differences between UTUC and UCB requires validation. Therefore, further investigations incorporating cohorts with larger sample sizes and functional validation are necessary to clarify the biological relevance of these findings.

### Malignant cells in MI tumours exhibit an enhanced immune-related features

**Figure 2:**
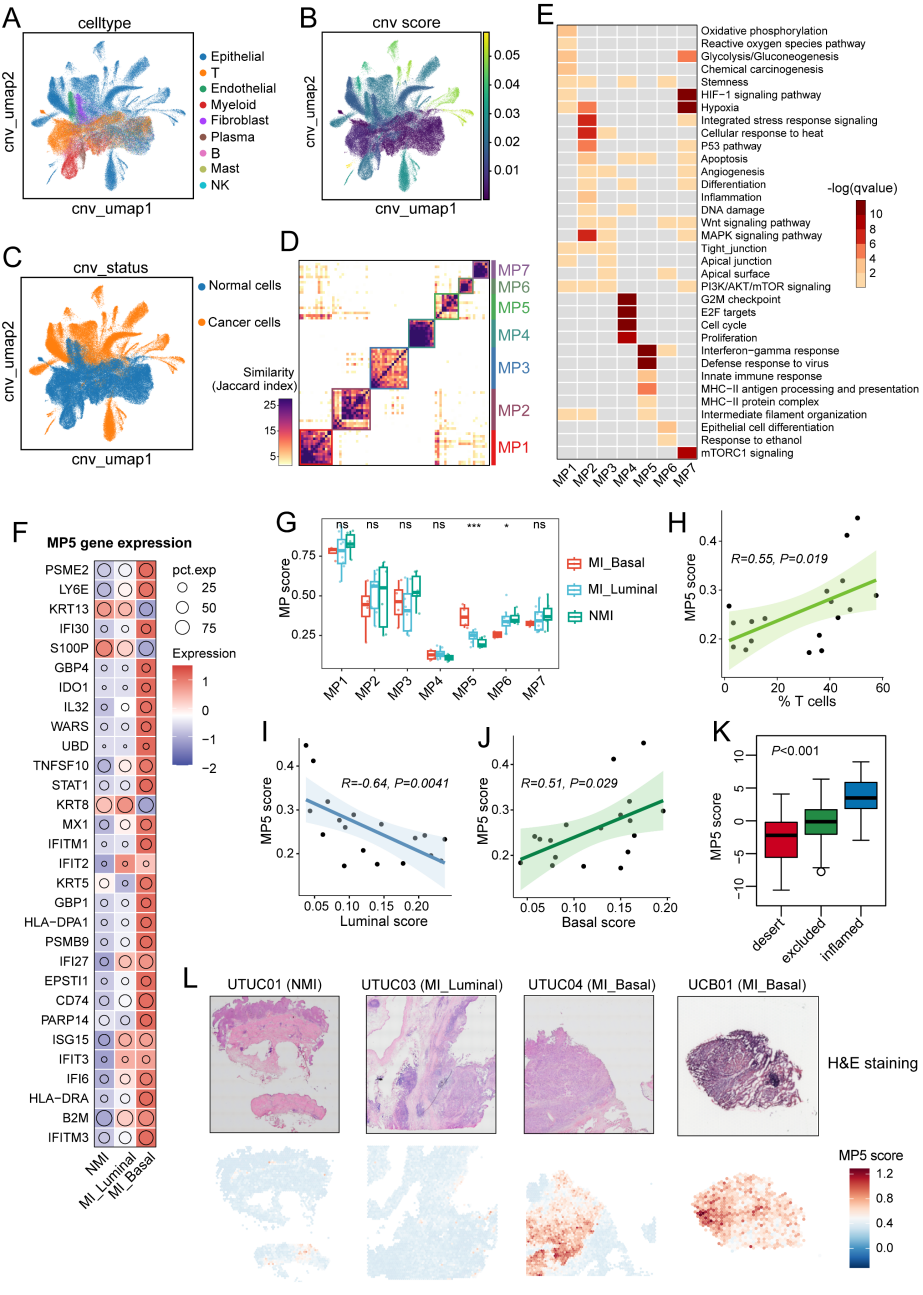
Identification and functional analysis of intratumoral malignant meta-programs in UC. (A) UMAP plot showing nine cell types identified from tumor samples. (B) UMAP plot highlighting copy number variation (CNV) scores across all cells. (C) UMAP visualization of CNV status across all cells. (D) Heatmap showing the Jaccard similarity index of robust NMF programs, hierarchically clustered into seven meta-programs (MPs). (E) Heatmap illustrating biological pathway enrichment of the seven MPs. (F) Bubble plot displaying the expression levels of 30 signature genes associated with MP5. (G) Bar plots comparing the scores of the seven MPs across different groups. Colored dots represent individual samples. Statistical significance is indicated by asterisks (*P ≤ 0.05, **P ≤ 0.01, ***P ≤ 0.001), and “ns” indicates no statistical difference. (H) Spearman correlations between MP5 scores and T-cell proportions in tumor samples. (I, J) Spearman correlations between MP5 scores and phenotype signature scores in tumor samples: (I) luminal scores; (J) basal scores. (K) Bar plot comparing MP5 scores across three immune subtypes (Inflamed, Excluded, and Desert) in the IMvigor210 cohort. (L) Spatial feature plots showing H&E-staining images (top) and MP5 signature scores (bottom) in tumor tissue sections across molecular subtype. Specifically, the UCB01 sample was obtained from the publicly available dataset (GSE171351).

Clusters of epithelial cells in tumour samples were highly dispersed, which indicates significant intertumoral heterogeneity (Figure 2A). We distinguished 49,698 cancer cells from epithelial cells based on the basis of the inferred CNV scores (Figure 2B, 2C, S2A, S2B). A consensus NMF algorithm was then applied to identify gene programs preferentially expressed in cancer cells across tumour samples, which resulted in 133 robust NMF programs. Clustering analysis retained seven meta-programs (MP1-7) that represent common expression patterns (Figure 2D). Each MP was characterized by a distinct signature of 30 genes (Table S5, and Figure S2C) and was annotated according to functional enrichment analysis (Figure 2E, S2D). The identified MPs are associated with various biological processes, including oxidative phosphorylation and metabolism (MP1), stress and hypoxia responses (MP2 and MP7), urothelium-related features (MP3 and MP6), and cell cycle/proliferation (MP4) (Figure 2E). MP5, characterized by immune-related features, includes genes involved in MHC-II antigen presentation (e.g., *CD74*, *HLA-DRA*, and *HLA-DPA1*) and the interferon response (e.g., *IFITM3* and *IFI6*). Notably, the majority of genes in MP5 were highly expressed in the MI group, particularly in MI basal phenotype samples (Figure 2F).

To investigate the relationships between MPs and tumour progression as well as tissue-specific features, we calculated the MP signature scores across all cancer cells and compared the average scores between tumour sample groups. Tumours in the MI group had significantly higher MP5 scores (*P* < 0.01) (Figure S2E), particularly in MI basal samples (*P* < 0.001) (Figure 2G), which suggests that the MP5 signature is associated with tumour progression. MP6, which is linked to luminal phenotype related genes (e.g., *SPINK1*, *UPK1*, and *UPK3*), had significantly lower scores in MI basal samples (*P* < 0.05) (Figure S2C and Figure 2G). However, no significant difference in MP scores was observed between UTUC and UCB (Figure S2F). Additionally, we found that the MP5 score were positively correlated with T-cell ratio (*R* = 0.55; *P* = 0.019), the myeloid cell ratio (*R*=0.62; *P*=0.0062), and the basal phenotype signature score (*R* = 0.51; *P* = 0.029), but negatively correlated with the luminal signature score (*R* = -0.64; *P* = 0.0041) (Figure 2H-2I, and Figure S2G-S2H). MP5 scores were highest in immune-inflamed tumours and lowest in immune-desert tumours of the IMvigor210 cohort (*P* < 0.001) (Figure 2K). Spatial analysis further revealed that most spots in the MI basal UC samples had high MP5 scores, wheras those in the NMI and MI luminal UC samples had lower scores (Figure 2L).

### Expansion of immunosuppressive T cells during UC progression

**Figure 3:**
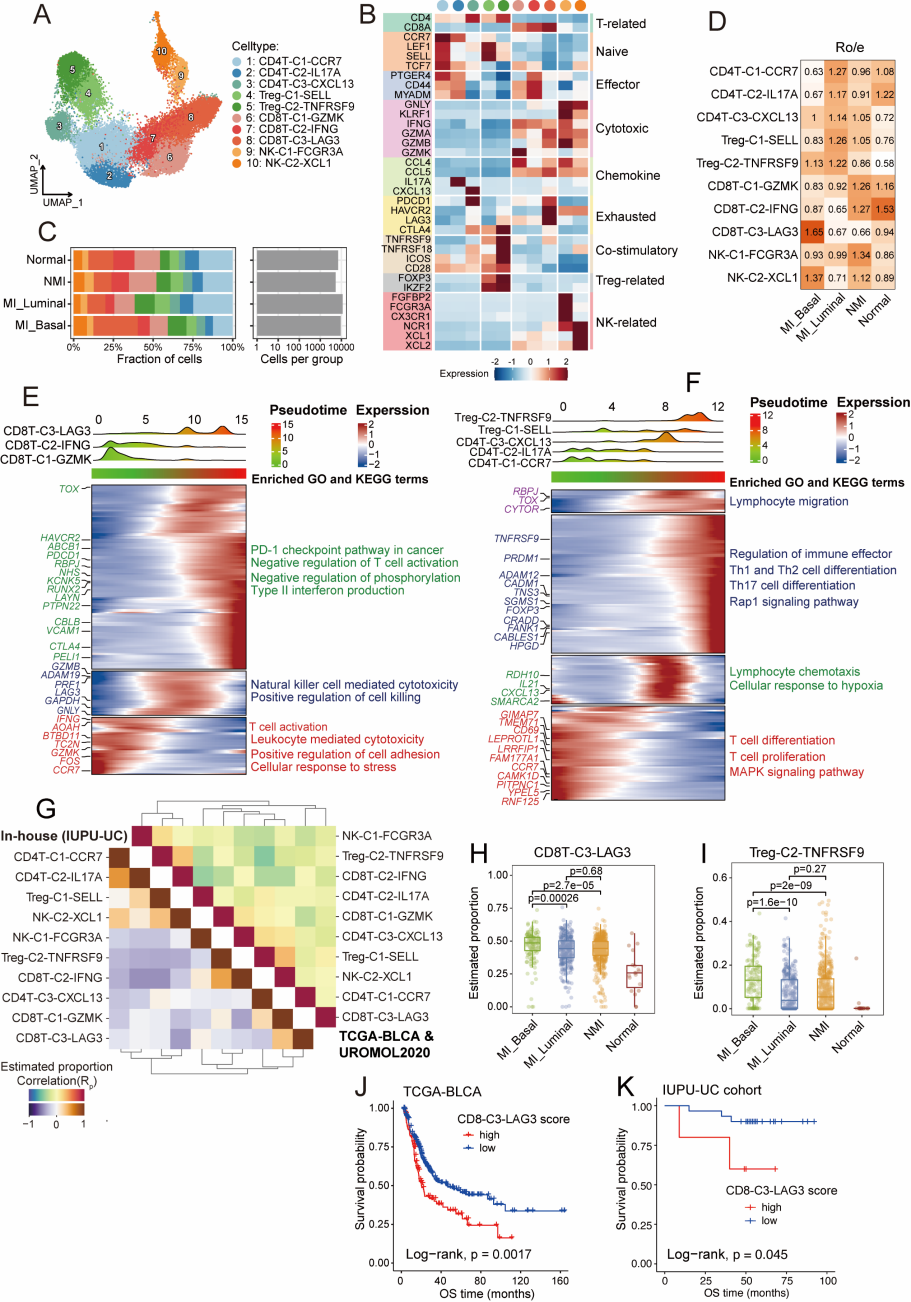
Diversity and dynamics of T and NK cells during UC progression. (A) UMAP plot of all T and NK cells, colored by ten distinct cell subsets. (B) Heatmap depicting the expression of function-associated signature genes used to define T and NK cells, with color intensity indicating normalized expression levels. (C) Cell fractions (as percentages) and cell numbers of T and NK cell subsets stratified by molecular subtypes. (D) Heatmap of R_o/e_ values for T and NK cell subsets across molecular subtypes. (E, F) Heatmaps displaying dynamic changes in gene expression of CD8^+^ T cells (E) and CD4^+^ T cells subsets (F). Normalized gene expression levels and pseudotimes were indicated by color gradients (upper). Gene expression profiles were hierarchically clustered (left), with enriched GO and KEGG pathway terms shown on the right. (G) Heatmap illustrating Spearman correlations among the relative abundances of T and NK cell subsets across the TCGA-BLCA, UROMOL2020, and in-house IUPU-UC cohorts. Cell abundances were inferred using the deconvolution algorithm BayesPrism. Correlation coefficients are represented by the color scale. (H, I) Box plots comparing the estimated infiltration proportions of CD8T-C3-LAG3 (H) and Treg-C2-TNFRSF9 (I) across the molecular subtypes. Colored dots represent individual samples. (J, K) Kaplan-Meier survival analysis of the overall survival (OS) of patients in the TCGA-BLCA (J) and in-house IUPU-UC (K) cohorts, stratified by high versus low signature scores of CD8T-C3-LAG3.

Tumour-infiltrating lymphocytes play dual roles in the tumour microenvironment: they mediate tumour cell recognition and cytotoxicity, thereby enhancing anti-tumor immunity, while specific subsets, such as regulatory T cells (Tregs), contribute to immune suppression and tumor immune evasion. We reclustered T cells and natural killer (NK) cells into ten major subtypes (Figure 3A) and annotated these subtypes on basis of reported function-associated signatures (Figure 3B). The subtypes included three CD4^+^ T cell clusters (CD4T-C1-CCR7, CD4T-C2-IL17A, and CD4T-C3-CXCL13), two Treg cell clusters (Treg-C1-SELL and Treg-C2-TNFRSF9), three CD8^+^ T cell clusters (CD8T-C1-GZMK, CD8T-C2-IFNG, and CD8T-C3-LAG3) and two NK cell clusters (NK-C1-FCGR3A and NK-C2-XCL1).

Additionally, we used the signatures of TCellSI 23 to assess the T-cell states, and reported that the regulatory signature scores of the Treg-C2-TNFRSF9 subtype were the highest (Figure S3A, Table S4). The Treg-C2-TNFRSF9 subtype was enriched in MI tumour samples, while the CD8T-C1-GZMK and CD8T-C2-IFNG clusters were predominantly found in NMI tumours and normal samples (Figure 3C and 3D). Pseudotime trajectory analysis revealed the diversification and developmental dynamics of CD8+ and CD4^+^ T cells (Figure 3E and 3F). The CD8T-C3-LAG3 subpopulation, characterized by high expression of exhausted markers such as PDCD1, HAVCR2, and LAG3 (Figure 3B), was located at the terminal end of the pseudotime and was enriched in pathways related to the PD-1 checkpoint, type II interferon production, and T-cell negative regulation. According to the results of R_o/e_ distribution analysis, the CD8T-C3-LAG3 subtype was found to be preferentially enriched in MI basal tumour samples (Figure 3D). The Treg-C2-TNFRSF9 subtype, which highly expresses costimulatory signatures (TNFRSF9, TNFRSF18, ICOS, and CTLA4), was found to be associated with the regulation of immune effector pathways and the Rap1 signalling pathway. These findings suggest that the immunosuppressive microenvironment may drive tumour progression towards MI. To validate the T-cell states across different tumour subgroups in the above analysis, we used the deconvolution algorithm BayesPrism 27 to analyse T-cell subtype abundance in bulk RNA sequencing data from the TCGA-BLCA and the UROMOL 2020 cohort, as well as the in-house IUPU-UC cohort. Interestingly, we detected a strong negative correlation between the frequency of CD8T-C3-LAG3 and CD8T-C2-IFNG in both cohorts (Figure 3G). The frequencies of CD8T-C3-LAG3 (*P* < 0.001) and Treg-C2-TNFRSF9 (*P* < 0.001) were significantly greater in MI basal tumour samples than in MI luminal and NMI samples (Figure 3H, 3I). In addition, KM analysis revealed that patients with a high degree of CD8T-C3-LAG3 infiltration exhibited worse overall survival (OS) in both the TCGA-BLCA cohort (*P* = 0.0017) (Figure 3J) and the IUPU-UC cohort (*P* = 0.045) (Figure 3K). No significant difference in prognosis was observed between patients with high and low levels of Treg-C2-TNFRSF9 infiltration in thee TCGA-BLCA cohort (*P* = 0.49) (Figure S3B), whereas patients with high levels of Treg-C2-TNFRSF9 infiltration had worse OS in the in-house UC cohort (*P* = 0.0079) (Figure S3D).

Currently, the classification of exhausted CD8^+^ T cells (CD8Tex) is not completely understood, as several studies have identified three distinct subsets in the tumour microenvironment: progenitor, intermediate, and terminally exhausted subsets 36. In this study, ROGUE index analysis 37 revealed lower cell purity in the CD8T-C3-LAG3 subset than in other CD8^+^ T cell subsets (Figure S3D), which indicates a high degree of heterogeneity among CD8Tex cells. To elucidate this heterogeneity, we further stratified CD8Tex cells into three subsets (Figure S3E). One cluster, which was characterized by high expression of *GZMK*, *CD44*, and *ICOS*, and the highest cytoTRACE scores (Figure S3F and S3G), was designated as CD8Tex-Prog, representing a progenitor-like state of CD8^+^ T cell exhaustion. The remaining two subsets exhibited upregulation of exhaustion-associated markers (Figure S3G). Among them, one subset that displayed markedly higher expression of terminal exhaustion markers, including *CTLA4*, *TIGIT*, *BATF*, *CXCL13* and* PRDM1*, and significantly elevated terminal exhaustion scores (*P* < 0.001), was defined as CD8Tex-Term (terminally exhausted cells) (Figure S3G and S3H). The final subset, characterized by an intermediate exhaustion state, was termed CD8Tex-Int. We observed that CD8Tex-Prog cells were relatively enriched in normal tissues, whereas CD8Tex-Int and CD8Tex-Term cells were more abundant in tumour samples (Figure S3I). Furthermore, KM survival curves revealed that patients with a high degree of CD8Tex-term infiltration had significantly worse OS in both the TCGA-BLCA cohort (*P*=0.0014) (Figure S3J) and the in-house cohort (*P*=0.049) (Figure S3K).

### Deciphering myeloid cell states associated with UC progression

**Figure 4:**
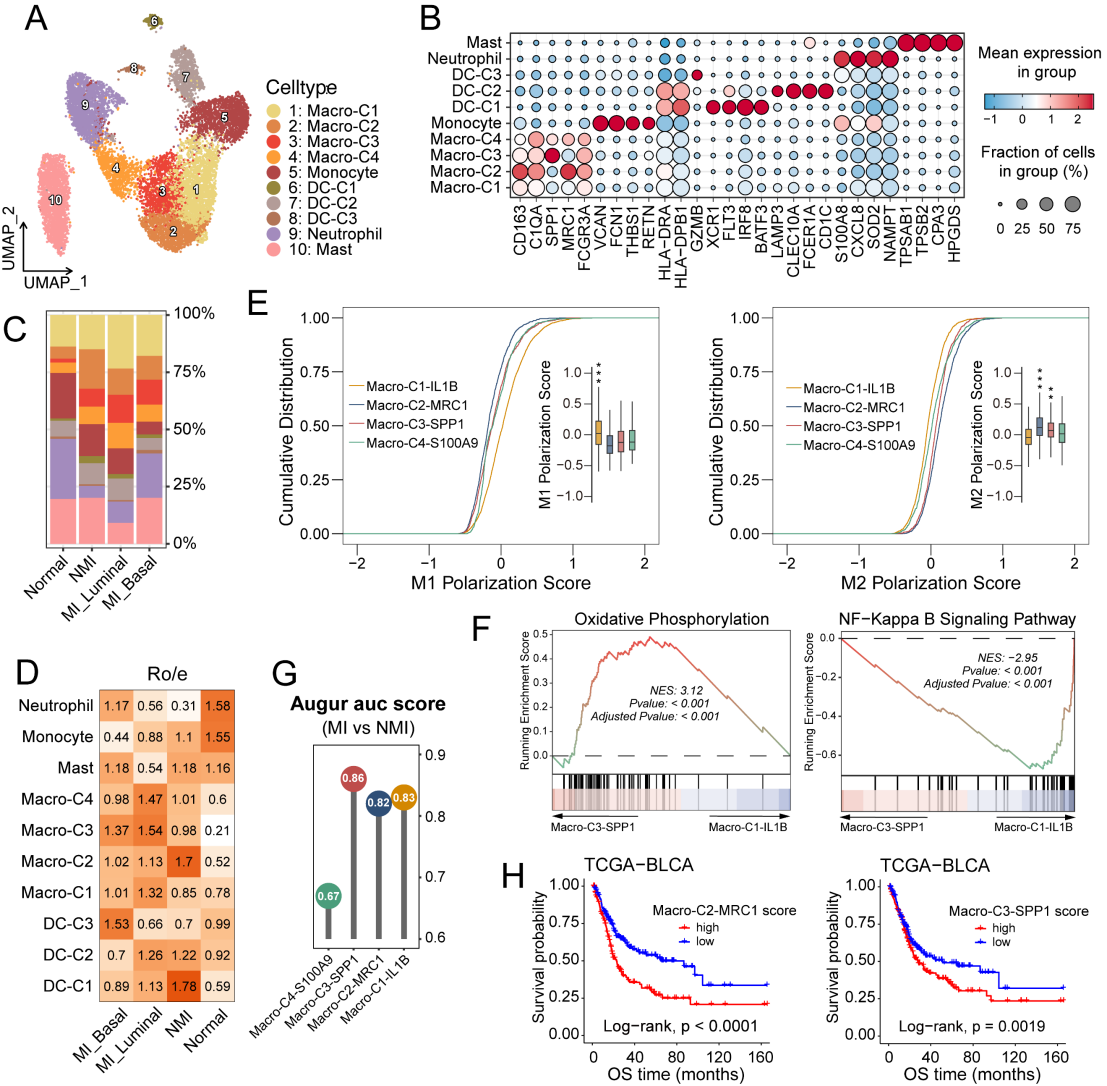
Characterization of subclusters and function profiles of myeloid cells in UC. (A) UMAP plot of myeloid cells, colored by ten subclusters. (B) Bubble plot showing differentially expressed genes across myeloid subsets. Dot size represents the proportion of cells expressing the gene, and color indicates the average normalized expression level. (C) Bar plot showing proportional abundances (percentages) of myeloid cell subsets across pathological and molecular subtypes. (D) Heatmap of R_o/e_ values for myeloid clusters across molecular subtypes. (E) Cumulative distribution plots of M1 and M2 polarization scores across four macrophage clusters, accompanying box plots show the score distributions per cluster. Statistically significant p-values are indicated by asterisks (**P ≤ 0.01, ***P ≤ 0.001). (F) Gene set enrichment analysis (GSEA) of pathways enriched in macro-C3-SPP1 cells compared to macro-C2-ILB cells. (G) Lollipop chart of the area under the curve (AUC) scores from Augur analysis across four macrophage subclusters, highlighting cell type prioritization between muscle-invasive (MI) and non-muscle-invasive (NMI) groups. (H) Kaplan-Meier survival curves for overall survival (OS) of patients in the TCGA-BLCA cohort, stratified by high versus low infiltration of macro-C2-MRC1 (left) and macro-C3-SPP1 (right). Statistical significance was determined using the log-rank test.

The increased infiltration of myeloid cells in MI tumour samples suggests that these cells may promote tumour progression (Figure 1E, 1F). Following reclustering analysis, we identified ten distinct myeloid subsets. On the basis of differentially expressed genes, these subsets were classified into five major categories: macrophages (macro) (C1-C4), monocytes, dendritic cells (DCs) (C1-C3), neutrophils and mast cells (Figure 4A, 4B). The myeloid subsets exhibited distinct distribution patterns across pathological and molecular subgroups. Cells in the DC-C1 cluster, characterized by high expression of *XCR1* and *IRF8*, were identified as conventional type 1 dendritic cells (cDC1s) and were predominantly enriched in NMI tumour samples. In contrast, cells in the DC-C2 cluster, which expressed high levels of *CD1c* and *CLEC10A*, corresponded to conventional type 2 dendritic cells (cDC2s), the distribution of which did not significantly differ across the subgroups (Figures 4C and 4D). Cells in the DC-C3 cluster, uniquely express *GZMB*, and along with neutrophils, were increased in MI basal tumour samples (Figure 4D).

Macrophage infiltration was elevated in tumour samples (Figure 4D), which highlights their pivotal role in tumorigenesis and tumour progression. The functional heterogeneity of tumour-associated macrophages (TAMs) is underscored by their distinct phenotypic subsets, commonly categorized into antitumour M1 (classically activated) and protumour M2 (alternatively activated) polarization states 38. In this study, we identified four TAM subclusters: Macro-C1-IL1B, Macro-C2-MCR1, Macro-C3-SPP1, and Macro-C4-S100A9 (Figures 4A, 4B and S4A). Analysis of M1 and M2 polarization signatures (Table S4) scores revealed that Macro-C1-IL1B had elevated M1 scores (*P* < 0.001), whereas Macro-C2-MCR1 (*P* < 0.001) and Macro-C3-SPP1 (*P* < 0.01) had higher M2 scores (Figures 4E and S4B). Among these subsets, Macro-C4-S100A9 demonstrated the greatest developmental potential (*P* < 0.001) (Figure S4C), potentially indicating a polarization-quiescent or M0-like state. Macro-C3-SPP1 was enriched in oxidative phosphorylation and PPAR signalling pathways, whereas Macro-C1-IL1B was predominantly associated with inflammatory pathways such as NF-κB and TNF signaling (Figures 4F, S4D, S4E). Augur 39 analysis revealed that Macro-C3-SPP1 was prioritized in MI tumour samples (Figure 4G). Furthermore, in the TCGA-BLCA cohort, increased infiltration of Macro-C3-SPP1 and Macro-C2-MCR1 was significantly correlated with reduced overall survival rates (*P* < 0.001 and *P* = 0.0019, respectively) (Figure 4H). Collectively, these findings suggest that the enrichment of Macro-C3-SPP1 may play a critical role in promoting muscle invasion in UC.

### Cellular heterogeneity of stromal cells in UC

**Figure 5:**
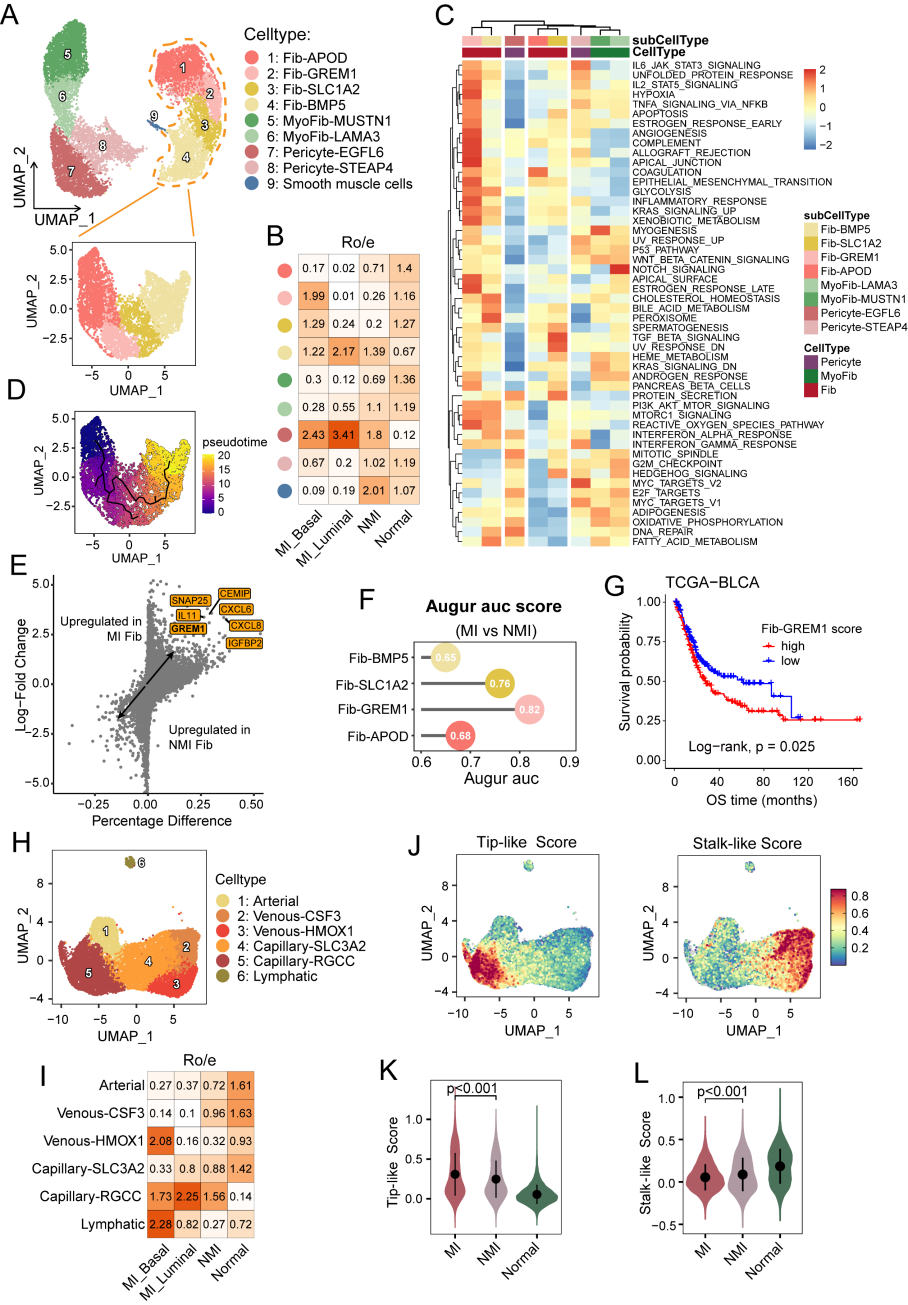
Cellular heterogeneity of stromal cells. (A) UMAP showing the COL1A1^+^ stromal cells, colored by nine subclusters. (B) Heatmap of R_o/e_ values for fibroblast subclusters across pathological and molecular subtypes. (C) Heatmap showing the GSVA enrichment scores for hallmark pathways across COL1A1^+^ stromal cell subclusters. (D) UMAP illustrating the developmental trajectories of fibroblasts across subclusters. (E) Volcano plot showing differentially expressed genes in fibroblasts between muscle-invasive (MI) and non-muscle-invasive (NMI) samples. (F) Lollipop chart showing area under the curve (AUC) scores from the Augur analysis, highlighting cell type prioritization among fibroblast subclusters between MI and NMI samples. (G) Kaplan-Meier survival curves for overall survival (OS) in the TCGA-BLCA cohort, stratified by high versus low infiltration of fib-GREM1. Statistical significance was determined using the log-rank test. (H) UMAP plot showing endothelial cells, colored by six subclusters. (I) Heatmap of R_o/e_ values for endothelial clusters across pathological and molecular subtypes. (J) UMAP showing endothelial cells, colored by tip-like and stalk-like scores. (K, L) Violin plots showing the tip-like (K) and stalk-like (L) scores across the normal, NMI and MI samples.

Stromal cells play crucial roles in the tumour microenvironment. To determine their cellular heterogeneity, a total of 29,573 stromal cells were extracted for reclustering. COL1A1^+^ stromal cells were categorized into four major clusters: cancer-associated fibroblasts (fib), identified by high expression of *PDGFRA*, were further divided into four subgroups (Figure 5A, S5A). Myofibroblasts (myoFib), defined by high *RGS5* expression 12, can be further categorized into two subtypes: myoFib-MUSTN1 and myoFib-LAMA3. Pericytes, characterized by high expression of *KCNJ8* 40, were further classified into two subsets: pericytes-EGFL6 and pericytes-STEAP4 (Figure 5A, S5A).

Fib-APOD, which was observed primarily in normal tissues (Figure 5B), was enriched in complement and coagulation pathways (Figure 5C), and was associated with the upregulation of genes associated with tissue regeneration and proliferation, including *APOD* and *IGF1* (Figures S5A, S5C). This subcluster closely resembled the *COL15A1*⁺ fibroblast progenitors described by Gao et al. 33. A bidirectional differentiation trajectory was observed in fibroblasts, which transitioned from a Fib-APOD phenotype to an inflammatory and interferon-regulated phenotype (Figure 5SB, 5D). Fib-BMP5 was found mainly in the tumour samples, and was characterized by the elevated expression of *SLC14A1*, *NRG1*, and *WNT5A* (Figures S5A and S5C). This subcluster was likely induced by interferon response signalling (Figure 5C) and has been reported to be linked to poor clinical outcomes 41. Fib-GREM1, which was enriched mainly in MI basal tumour samples, exhibited characteristics associated with inflammatory responses and epithelial-mesenchymal transition, which highlights its immunoregulatory functions. Moreover, this subcluster underwent metabolic reprogramming characterized by increased hypoxia and glycolysis, reflecting its specialized adaptations to the tumour microenvironment. Notably, elevated *GREM1* expression has been observed in fibroblasts across various carcinomas, where it contributes to tumour cell proliferation and invasion 42. Consistently, *GREM1* expression was upregulated in fibroblasts from MI samples, and Augur analysis confirmed the preferential enrichment of Fib-GREM1 in MI tumors (Figure 5E and 5F). Additionally, a higher Fib-GREM1 score was significantly associated with reduced overall survival rates (*P* = 0.025) (Figure 5G).

Endothelial cells (ECs) and mural cells are the main components of the vasculature. Angiogenesis, a critical hallmark of cancer, is driven by the migration of tip cells and the proliferation of endothelial progenitor cells, which promote the sprouting of new blood vessels into the tumour microenvironment 25. In our analysis, ECs were categorized into lymphatic and vascular ECs, and the latter were further divided into one arterial, two venous (venous-CSF3 and venous-HMOX1), and two capillary (capillary-SLC3A2 and capillary-RGCC) EC subsets (Figure 5H and S5D). Notably, compared with normal tissues, capillary-RGCC ECs were enriched in tumour samples and exhibited upregulated expression of genes associated with the G2M checkpoint, the mitotic spindle, and Wnt/β-catenin signalling (Figure 5I and S5E). Interestingly, we found that lymphatic ECs were enriched only in MI basal tumour samples (Figure 5I). To further elucidate the role of ECs in muscle-invasive progression, we assessed EC phenotypes based on the tip and stalk cell gene signatures 25 (Figure 5J, Table S4). Among the EC subsets, capillary-RGCC ECs displayed the highest tip-like scores but the lowest stalk-like scores (*P* < 0.001 and *P* = 0.0019, respectively) (Figure S5F and S5G). Moreover, MI tumour tissues were significantly enriched in tip-like ECs (*P* < 0.001), whereas ECs in NMI tumuor tissues exhibited higher stalk-like scores (*P* < 0.001) (Figure 5K and 5L), which suggests a phenotypic shift in ECs associated with progression to MI UC.

### Interferon-γ signalling drives MHC-II expression in cancer cells

**Figure 6:**
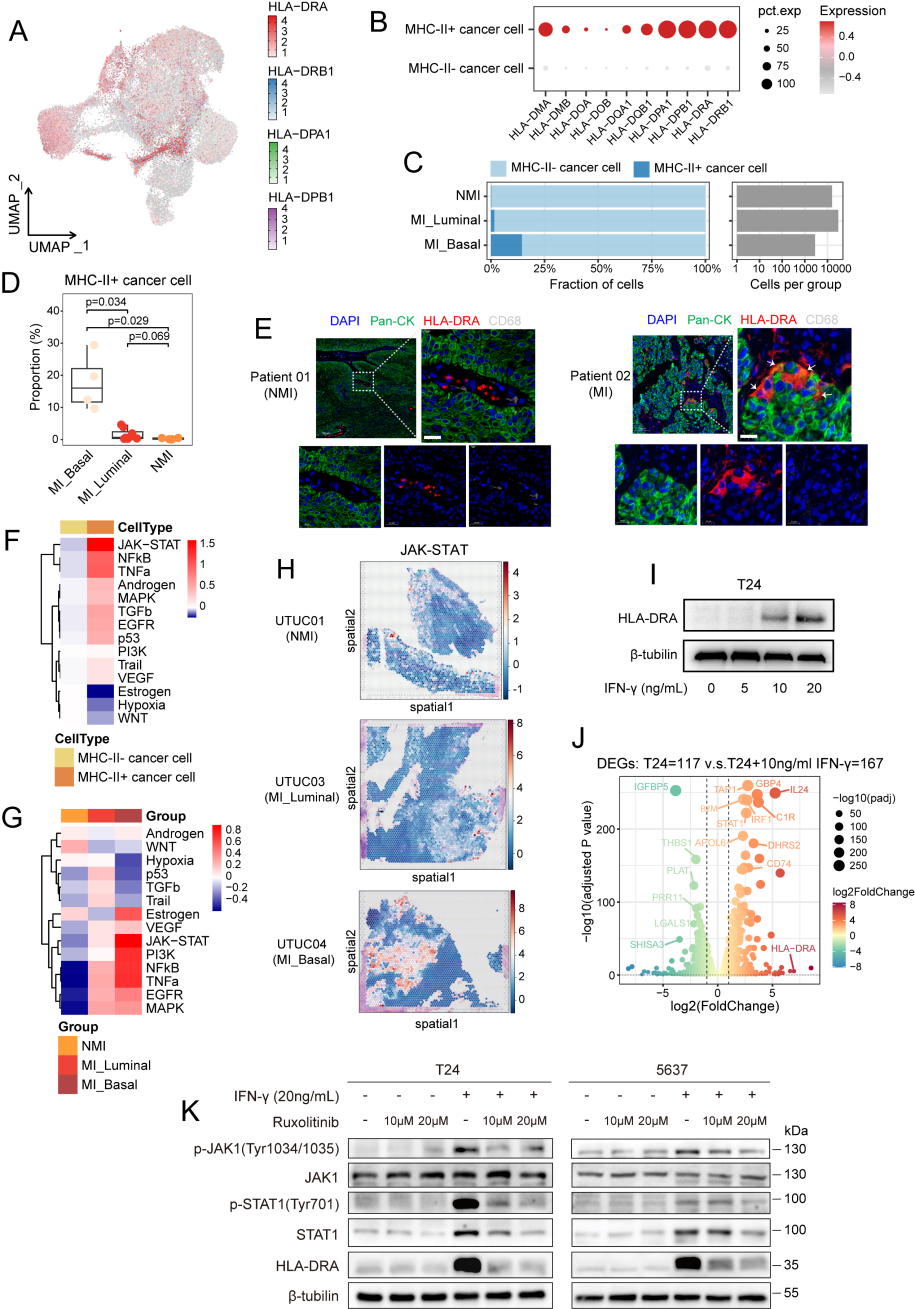
IFN-γ signalling drives the expression of MHC-II on cancer cells. (A) UMAP visualization of cancer cells, colored by the expression levels of four MHC-II genes. (B) Dot plot showing expression of all MHC-II molecule genes in MHC-II^+^ and MHC-II^-^ cancer cells. (C) Proportional abundance (percentage) of cancer cell subsets across pathological and molecular subtypes. (D) Comparison of the average percentage of MHC-II^+^ cancer cells across pathological and molecular subtypes. (E) Immunofluorescence staining of nuclei (DAPI, blue), pan-cytokeratin (pan-CK) (green), HLA-DRA (red), and CD68 (gray) in non-muscle-invasive (NMI) and muscle-invasive (MI) UC tumors. Colocalization of pan-CK and HLA-DRA appears yellow in merged images (white arrows). (F, G) Heatmaps of pathway activity scores from PROGENy analysis in cancer cells, grouped by MHC-II expression (F) and by pathological and molecular subtype (G). (H) PROGENy analysis of JAK/STAT pathway activity in spatial transcriptomic sections. (I) Western blot analysis of HLA-DRA expression in T24 cells treated with IFN-γ (0, 5, 10, 20 ng/mL) for 72 hours. (J) Volcano plot showing differentially expressed genes between T24 cells treated with or without IFN-γ (10 ng/ml). (K) Western blot analysis of T24 (left) and 5637 (right) cell lines demonstrates that IFN-γ-induced HLA-DRA expression is mediated through the JAK1/STAT1 signaling. IFN-γ-induced increases in JAK1 and STAT1 phosphorylation, as well as the expression of total STAT1 and HLA-DRA were suppressed by treatment with the JAK1/2 inhibitor ruxolitinib.

Cancer cells harbouring MP5 status exhibited high expression of genes associated with MHC-II antigen presentation and the interferon response. To further investigate the epithelial-immune dual feature of these cells, we stratified them into MHC-II^+^ and MHC-II^-^ subgroups according to their coexpression of four canonical MHC-II-related genes (*HLA-DRA*, *HLA-DRB1*, *HLA-DPA1*, and *HLA-DPB1*) (Figure 6A). Cells that exhibited detectable expression (expression level > 0) of all four genes were classified as MHC-II^+^, while the remaining cells were designated MHC-II^-^. This binary classification yielded a robust separation: MHC-II⁺ cancer cells consistently expressed all the selected MHC-II genes, whereas MHC-II⁻ cells expressed almost none of these genes (Figure 6B). As expected, MHC-II^+^ cancer cells exhibited high expression of MP5 and basal phenotype gene signatures but low expression of luminal phenotype gene signatures (Figure S6A-C). The proportion of MHC-II^+^ cancer cells was the lowest in the NMI group but it was significantly greater in the MI basal group (*P*=0.029) (Figure 6C and 6D). Differential gene expression and gene ranking analyses revealed that the expression of *CD74*, *HLA-DRA*, *HLA-DRB1*, and *FXYD3* was elevated in MHC-II⁺ cancer cells, whereas the expression of *LOXL2* and *DLL4* was increased in MHC-II⁻ cancer cells (Figure S6D).

To validate the dual epithelial-immune feature of MHC-II^+^ cancer cells, we performed multiple immunofluorescence staining in UC tissues and confirmed the coexpression of epithelial markers (pan-CK) and HLA-DRA in single cancer cells from MI tumours (Figure 6E). As professional antigen-presenting cells, dendritic cells, B cells, and macrophages constitutively express MHC-II molecules along with the classical costimulatory molecules such as *CD80* and *CD86*, both of which are essential for CD4^+^ T-cell activation 43. Therefore, although they express MHC-II molecules, MHC-II⁺ cancer cells lack *CD86* expression (*P* < 0.001) (Figure S6E), rendering them incapable of effectively activating CD4⁺ T cells.

To explore the regulatory mechanisms underlying the generation of MHC-II^+^ cancer cells, we performed pySCENIC analysis. The results revealed that key transcription factors of the IFN-γ signalling pathway, including STAT1 and IFR1, exhibited increased activity in MHC-II^+^ cancer cells (Figure S6F). PROGENy analysis revealed that JAK/STAT signalling was the most prominently activated pathway in MHC-II⁺ cancer cells (Figure 6F). In addition, cancer cells within the MI basal subgroup demonstrated the highest level of JAK/STAT pathway activity among all the subgroups (Figure 6G). Spatial transcriptomic analysis confirmed elevated JAK/STAT pathway activity in MI basal tumors (Figure 6H). In addition to IFN-γ response-associated genes (*JAK1*, *STAT1* and *STAT3*), *CIITA*, a master regulator of MHC-II gene expression, was highly expressed in MHC-II⁺ cancer cells (Figure S6G, S6H). Notably, these cells exhibited greater differentiation potential than did their MHC-II⁻ counterparts (*P* < 0.001) (Figure S6I), which suggests a link between IFN-γ signalling and cancer cell plasticity. Given these findings, we hypothesized that IFN-γ-mediated JAK/STAT activation drives MHC-II⁺ cancer cell induction. In support of this finding, we observed that CD8⁺ T cells in MI tumours, especially those in the MI basal group, exhibited elevated IFNG expression (Figure S6J). This finding was further validated in CD8⁺ T-cell subsets (CD8T-C2-IFNG cells) (Figure S6K). These findings suggest that IFN-γ secretion by CD8⁺ T cells may promote MHC-II expression in cancer cells, thus shaping their immune interactions and differentiation trajectories.

We evaluated the expression levels of MHC-II molecules across 37 bladder cancer cell lines. Our results revealed that most of these cell lines exhibited low MHC-II expression (Figure S7A). Among them, T24 and 5637—both classified as the basal subtype—showed minimal expression of HLA-DRA. However, *in vitro* stimulation with IFN-γ at concentrations above 10 ng/mL significantly upregulated *HLA-DRA* expression in these cell lines (Figures 6I and S7B). Transcriptomic analysis further confirmed that IFN-γ stimulation activated relevant signalling pathways, including the IFN-γ response and the JAK/STAT pathway, leading to increased expression of MHC-II-related molecules such as *HLA-DRA*, *CD74*, *IRF1*, and *STAT1* (Figures 6J, S7C-S7G). To further validate that IFN-γ-induced MHC-II expression is mediated by the JAK/STAT pathway, we treated T24 and 5637 cells with the selective JAK1/2 inhibitor ruxolitinib. Western blot analysis revealed that IFN-γ stimulation increased the phosphorylation of JAK1 and STAT1, as well as the total STAT1 protein level, whereas these effects were markedly attenuated by treatment with ruxolitinib (Figure 6K).

### MHC-II⁺ cancer cells predict poor prognosis and contribute to tumour progression

**Figure 7:**
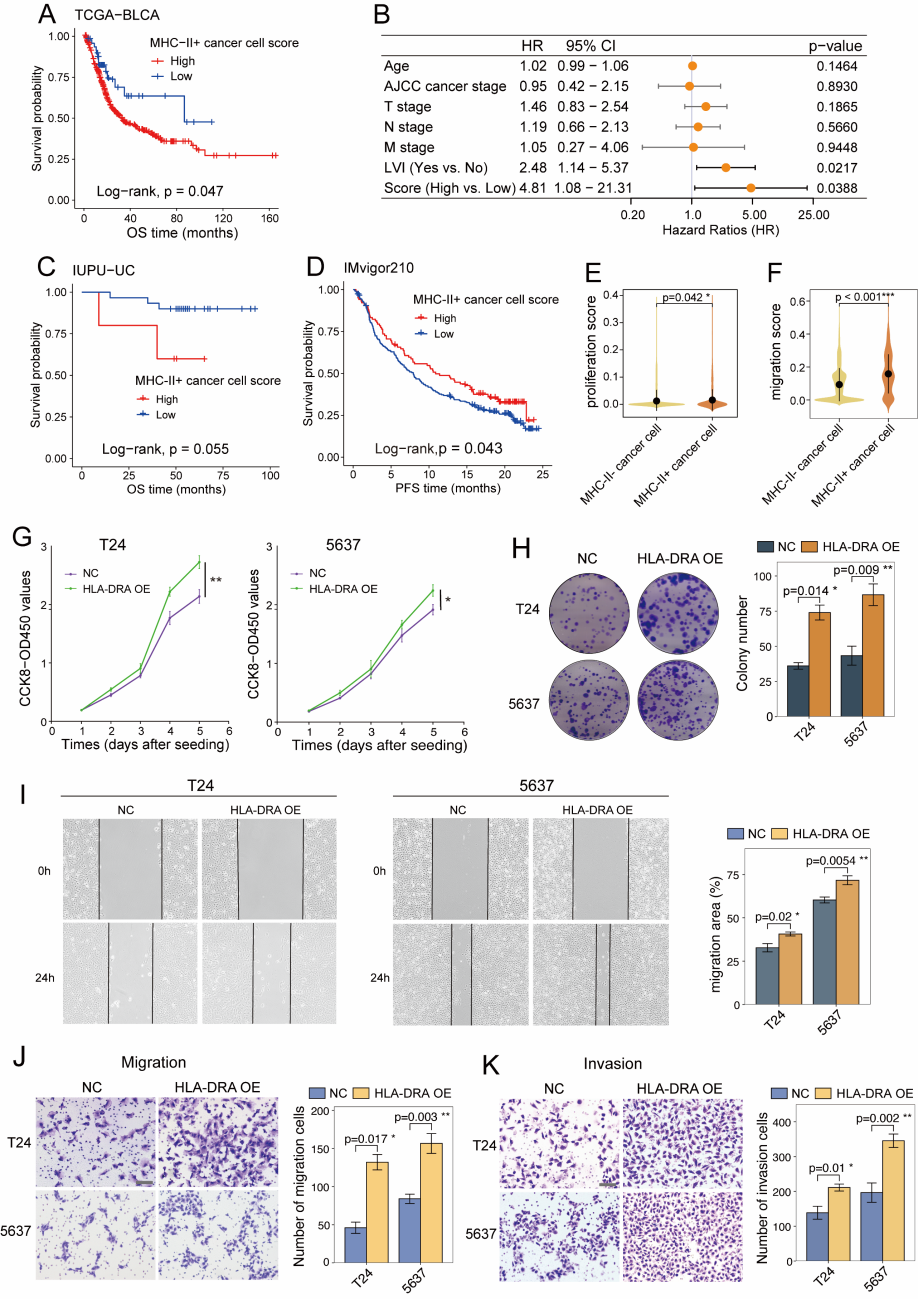
MHC-II^+^ cancer cells predict poor prognosis and promote tumor aggressiveness. (A) Kaplan-Meier survival curves for overall survival (OS) of patients in the TCGA-BLCA cohort, stratified by high versus low MHC-II^+^ cancer cell score. (B) Multivariate cox regression analysis of MHC-II^+^ cancer cells signature score and clinicopathologic factors in the TCGA-BLCA cohort, identifying lymphovascular invasion (LVI) and high MHC-II⁺ cancer cell scores as independent risk factors. (C) Kaplan-Meier curves of OS in the IUPU-UC cohort stratified by high versus low MHC-II⁺ cancer cell signature scores. (D) Kaplan-Meier curves of progression-free survival (PFS) in the IMvigor210 cohort stratified by high versus low MHC-II⁺ cancer cell signature scores. (E, F) Violin plots showing the proliferation (E) and migration (F) signature scores across cancer cell subsets in the scRNA-seq dataset. (G, H) CCK8 proliferation assay (G) and colony formation assay (H) showing the proliferation ability of T24 and 5637 cells with HLA-DRA overexpression. (I-K) Wound healing (I), Transwell migration (J) and Transwell invasion (K) assays demonstrating enhanced migration and invasion abilities of T24 and 5637 cells with HLA-DRA overexpression. scale bar, 50 μm. AJCC, American Joint Committee on Cancer.

We next assessed the prognostic relevance of MHC-II⁺ cancer cells in UC patients. KM analysis of the TCGA-BLCA cohort revealed that patients with a high MHC-II⁺ cancer cell signature score had significantly shorter OS (P = 0.047; Figure 7A). Multivariate Cox regression analysis revealed both lymphovascular invasion (LVI) (HR = 2.48; 95% CI: 1.14-5.37; *P* = 0.022) and a high MHC-II⁺ cancer cell signature score (HR = 4.81; 95% CI: 1.08-21.31; *P* = 0.039) as independent predictors of poor prognosis (Figure 7B). However, no significant association between the MHC-II⁺ cancer cell signature score and OS was observed in the IUPU-UC cohort based on either KM or multivariate Cox analysis (Figure 7C and S8A), which may be due to the limited sample size. In the IMvigor210 cohort, a high MHC-II⁺ score was associated with shorter OS without reaching statistical significance (*P* = 0.11; Figure S8B), but significantly correlated with better progression-free survival (*P* = 0.043; Figure 7D). Furthermore, although the difference was not statistically significant, higher MHC-II⁺ scores were observed in responders to anti-PD-L1 therapy than in non-responders (Figure S8C, D). These findings suggest a potential role for MHC-II⁺ expression as a predictive biomarker for immunotherapy, which warrants further investigation.

Given the prognostic impact of MHC-II⁺ cancer cells, we sought to characterize their distribution and function. Cell type deconvolution analysis revealed a significant enrichment of MHC-II⁺ cancer cells in MI basal tumours (*P*<0.001; Figure S8E). Functionally, MHC-II⁺ cancer cells exhibited higher proliferation (*P* = 0.042) and migration (*P* < 0.001) signature scores (Figures 7E and 7F; Table S4). Consistently, overexpression of *HLA-DRA* increased the proliferative capacity of T24 and 5637 cancer cells, as confirmed by CCK-8 (Figure 7G) and colony formation assays (Figure 7H). Moreover, as shown in wound healing (Figure 7I) and Transwell assays (Figures 7J and7K), *HLA-DRA* overexpression significantly promoted cancer cell migration and invasion. Taken together, these results suggest that elevated MHC-II expression on cancer cells may facilitate tumour progression.

### MHC-II⁺ cancer cells form spatial niches with T cells and SPP1^+^ macrophages

**Figure 8:**
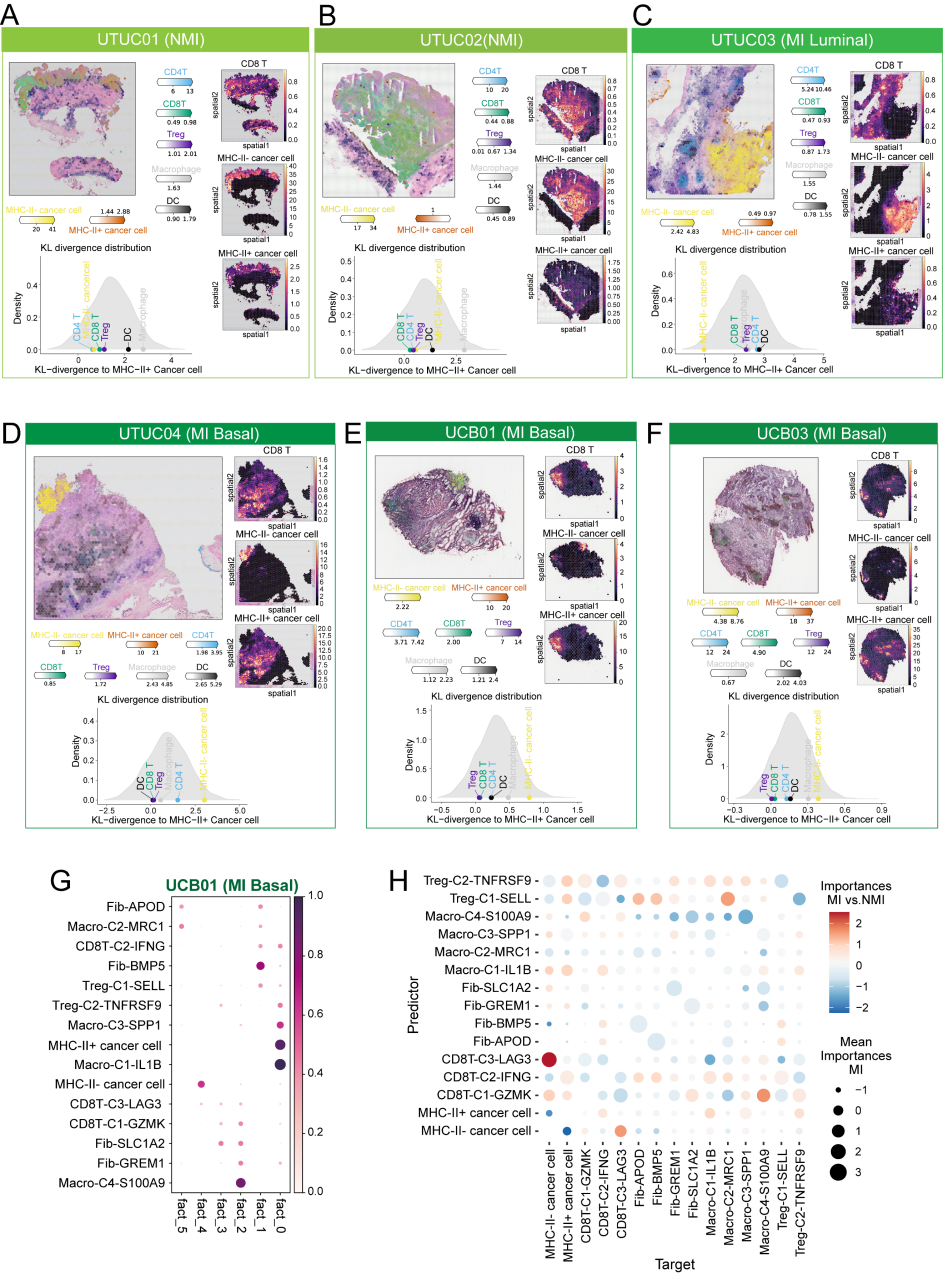
Spatial colocalization of MHC-II⁺ cancer cells with immune cells. (A-F) Spatial mapping of major cell types (left) and individual cell subsets (right) onto transcriptomics slides from UTUC (A-D) and UCB (E-F) tissues using cell2location, with color intensity indicating cell abundance. Below each panel (A-F), density plots display Kullback-Leibler (KL) divergence distributions, reflecting the degree of spatial association between MHC-II⁺ cancer cells and immune cell subsets. Each immune subset is represented by a dot along the KL axis; a position closer to zero indicates stronger colocalization with MHC-II⁺ cancer cells. Specifically, the UCB01 and UCB03 samples (E and F) were obtained from the publicly available dataset (GSE171351). (G) Identification of cell compartments using non-negative matrix factorization (NMF) in UCB01 tumor sections. Normalized weights of each cell type across NMF components are shown, with color intensity indicating the weight values. (H) MISTy-based estimation of cell cluster cooccurrence within spots across all slides, comparing muscle-invasive (MI) and non-muscle-invasive (NMI) UC samples.

To further assess the spatial relationships of MHC-II⁺ cancer cells, we employed the cell2location model along with cell-type specific expression profiles derived from our scRNA-seq dataset to deconvolute cell-type abundances across eight UC tissue sections (Table S2). Our analysis revealed that MHC-II⁺ cancer cells were less frequently observed in NMI and MI luminal samples (Figure 8A-C), whereas their distribution was markedly increased in MI basal samples (Figure 7D-F). Notably, MHC-II⁺ cancer cells preferentially colocalized with CD8⁺ T cells in MI basal samples (Figure 7D-F), which suggests that CD8⁺ T cells may play a pivotal role in driving the production of MHC-II⁺ cancer cells. To quantitatively assess the spatial relationships between cell subsets, we applied the Kullback-Leibler (KL) divergence analysis 33. Both density plots and heatmaps consistently revealed that MHC-II⁺ cancer cells exhibited greater spatial similarity and pronounced colocalization with CD8⁺ T cells and Tregs in MI basal tumours, as reflected by lower KL divergence values (Figure 8A-F and S9A-F).

In the NMI and MI luminal phenotype sections, immune cells were predominantly localized at the periphery of cancer cell regions (Figure 8A-C). However, in MI basal phenotype tissues, immune cells infiltrated deeper into the central regions of cancer cells (Figure 8D-F). We then applied NMF to the cell subcluster abundances inferred from cell2location across all tissue sections to identify spatial co-occurrence patterns, which provided insights into potential cellular interactions. Our analysis revealed that MHC-II⁺ cancer cells colocalized with CD8T-C2-IFNG, macro-C3-SPP1, and Treg-C2-TNFRSF9 in MI basal sections (Figure 8G, S9G-K).

To further investigate the colocalization dynamics within individual spots as well as across adjacent spots, we utilized the MISTy algorithm to analyse the spatial neighbourhood of cell subclusters across all tissue slides. Comparing the MI UC slides with the NMI UC slides, we observed that MHC-II⁺ cancer cells were indeed predicted to be colocalized with CD8T-C2-IFNG, macro-C3-SPP1, and Treg-C2-TNFRSF9, and vice versa (Figure 8H). However, in the NMI UC slides, we observed a notable decrease in the association between CD8T-C2-IFNG and Treg-C2-TNFRSF9 (Figure 8H). The observed shift in spatial associations in MI tissue slides may suggest alterations in immune cell dynamics that contribute to the distinct tumour microenvironment characteristics of these subtypes.

### MHC-II⁺ cancer cells shape the immunosuppressive landscape in UC

**Figure 9:**
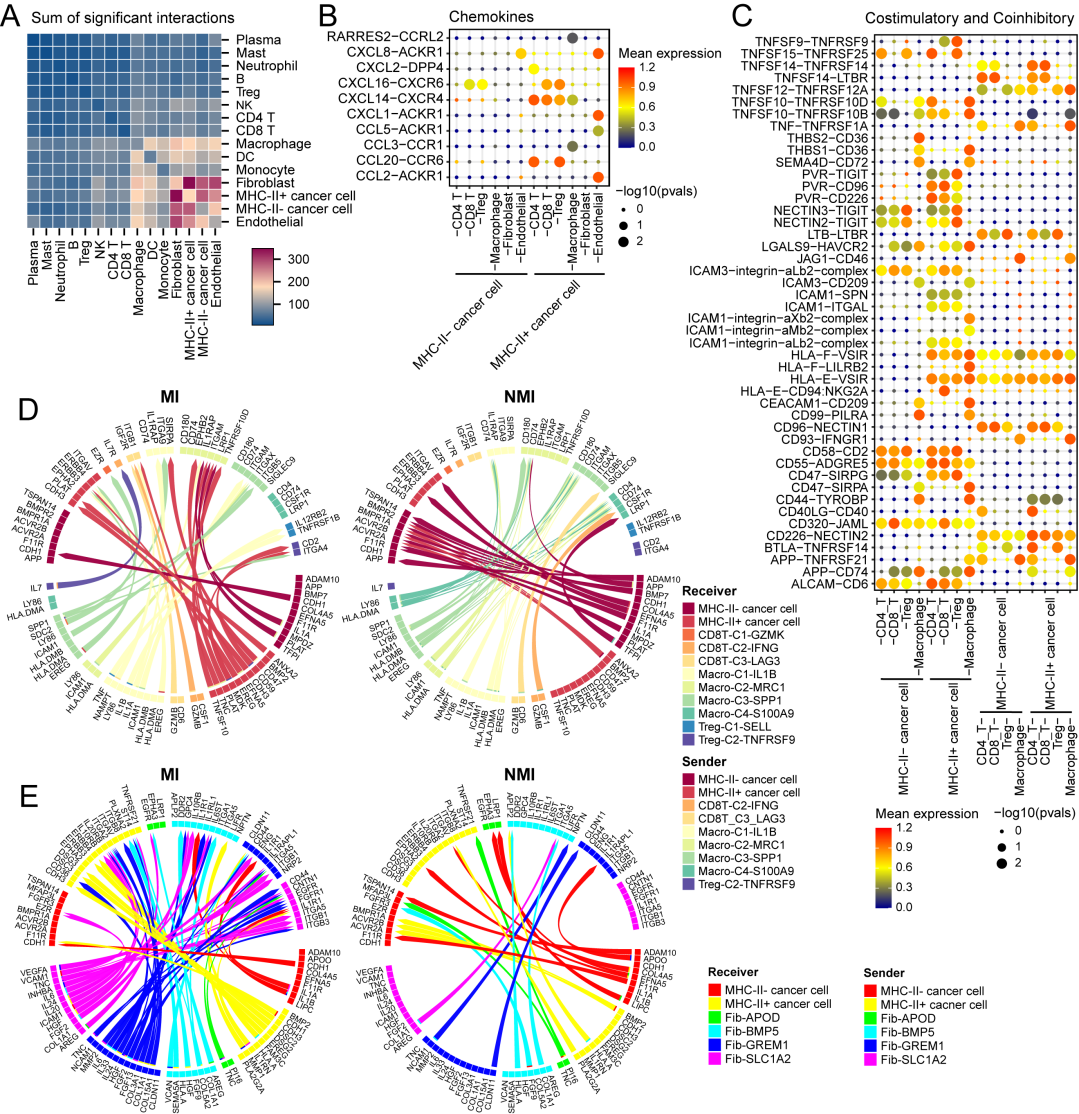
Interaction networks between MHC-II^+^ cancer cells and immune cells/fibroblasts. (A) Heatmap showing the number of the ligand-receptor pairs inferred by CellPhoneDB among major cell subsets. (B, C) Bubble plots showing ligand-receptor interactions involved in chemokine signaling (B) and costimulatory/coinhibitory molecules between cancer cells, T cells, macrophages, fibroblasts, and endothelial cells. Circle size indicates the -log_10_(p-value), and color represents the mean expression level. (D, E) Chord diagrams illustrating differential ligand-receptor interactions between MHC-II^+^ cancer cells and immune cells (D) or fibroblasts (E), in muscle-invasive (MI) versus non-muscle-invasive (NMI) UC samples. Arrowheads indicate interaction direction from sender to receiver cell; arrow color corresponds to the ligand-expressing sender cell type.

To investigate the role of MHC-II⁺ cancer cells in the tumour microenvironment, we used CellPhoneDB to analyse their interaction networks with various identified cell subtypes in our study. MHC-II⁺ cancer cells significantly interact with fibroblasts, macrophages, endothelial cells, and T cells (Figure 9A). Chemokines such as *CXCL14*, *CXCL16*, and *CCL20*, expressed by MHC-II⁺ cancer cells, mediate interactions with exhausted CD8⁺ T cells and Tregs (Figure 9B, S10A), and contribute to the immunosuppressive microenvironment 44, 45. Additionally, the interactions between chemokines, including *CXCL1*, *CXCL8*, and *CCL2*, and *ACKR1*, expressed by endothelial cells, were notably greater in MHC-II⁺ cancer cells than in MHC-II⁻ cancer cells (Figure 9B and S10A). *ACKR1* acts as a decoy receptor for these chemokines, promoting angiogenesis and enhancing pro-malignant effects 46. Furthermore, the regulatory network of MHC-II⁺ cancer cells revealed interactions between PVR and coinhibitory receptors including *TIGIT* and *CD96*, which induce CD8^+^ T-cell exhaustion and Treg activation, and subsequently facilitate immune evasion 47. The increased expression of *CD47* on MHC-II⁺ cancer cells further promoted interactions with *SIRPα* on macrophages, to a greater extent than that observed in MHC-II⁻ cancer cells (Figure 9C, S10B, S10C). This interaction inhibits phagocytosis and prevents the engulfment of cancer cells 48. Similar interactions were observed with *SIPRγ* on exhausted CD8⁺ T cells and Tregs (Figure S10B).

Next, we used MultiNicheNet to compare the cell‒cell interaction between the cell components of the MI and NMI groups. In the MI group, we detected significant interactions between macro-C3-SPP1 and MHC-II⁺ cancer cells via the EREG/ERBB4 axis (Figure 9D, S10D), which activates downstream MEK/ERK and PI3K/AKT signalling pathways, promoting cell proliferation and survival 49. In addition, we observed increased interactions between macro-C3-SPP1 and Treg-C2-TNFRSF9 through the *SPP1-ITGA4* axis in the MI group (Figure 9D, S10D), which could facilitate the adhesion and migration of activated Tregs within the tumour microenvironment, potentially contributing to immune suppression and tumour progression 50. Furthermore, in the MI group, fib-GREM1 was observed to significantly interact with MHC-II⁺ cancer cells through the *COL1A1-ITGA2* and *IL24-IL20RB* axes (Figure 9E), both of which are known to promote cancer cell stemness and support tumour aggressiveness.

## Discussion

In this study, we utilized a large-scale integrated scRNA-seq dataset to systematically characterize the intratumoral cellular heterogeneity and intertumour differences in UC, and we provide a comprehensive analysis of diverse cell populations and their functional states. A recent scRNA-seq study has demonstrated that although UTUC and UCB share similar cellular compositions, they harbour distinct functional cell subsets 15. Notably, UTUC is associated with a unique immunosuppressive microenvironment characterized by CD8^+^ T-cell exclusion and M1 macrophage expansion. However, these findings were limited by insufficient consideration of tumour stage variations and a constrained sample size, as only three UTUC cases were analysed, which may have affected the robustness and generalizability of the conclusions. Consistently, our analysis revealed a comparable distribution of major cell types between UCB and UTUC. However, the upregulation of the expression of genes associated with immunosuppression, such as *CTLA-4*, was not observed in UTUC. These differences between UTUC and UCB at single-cell resolution highlight the need for further investigations through larger-scale studies to validate these observations and determine their clinical implications.

Cancer cell plasticity enables phenotypic and functional shifts that drive tumor initiation, progression, metastasis, and therapeutic resistance 51. Our analysis revealed an epithelial-immune hybrid gene expression program (MP5) in cancer cells, characterized by the coexpression of interferon response genes and MHC-II components (Figure 2F). This program was positively associated with T-cell infiltration, the basal tumour phenotype, and an immune-inflamed microenvironment. Notably, this epithelial-immune dual feature was predominantly observed in cancer cells of MI UC tumors, particularly those of the basal subtype, and no significant differences were observed between UTUC and UCB.

Furthermore, we dichotomized cancer cells according to their MHC-II expression status, and revealed that the transcriptomic profile of MHC-II⁺ cancer cells was consistent with that of MP5 (Figure S6B). While MHC-II molecules are typically expressed on professional antigen-presenting cells, such as macrophages and DCs, their aberrant expression on cancer cells has been increasingly recognized. However, the functional implications of cancer cell-intrinsic MHC-II expression remain controversial. Although previous studies have linked cancer cell-specific MHC-II expression to a favourable prognosis in multiple cancer types including colon cancer 52, breast cancer 53, and melanoma 54, recent scRNA-seq analyses have suggested a contrasting role. Jin et al. 55 reported that HLA-DR^hi^ tumour cells in nasopharyngeal carcinoma contributed to CD8⁺ T-cell exhaustion and tumour progression by upregulating co-inhibitory receptors on infiltrating T cells. Similarly, Lei et al. 56 reported that MHC-II⁺ cancer cells induced Treg expansion while reducing CD4⁺ effector T cells in tumour-draining lymph nodes of patients with breast cancer, thereby facilitating metastasis and immune evasion. Another scRNA-seq study 57 demonstrated that MHC-II expression in alpha-fetoprotein-positive hepatocellular carcinoma is associated with immune dysfunction, including T-cell exhaustion and the accumulation of tumour-promoting macrophages.

We investigated the generation of MHC-II^+^ cancer cells and their role in the tumour microenvironment of UC. Our analysis revealed an upregulation of the IFN-γ response and JAK/STAT pathway in MHC-II⁺ cancer cells (Figure 6F, S6H). IFN-γ, which is typically produced by NK cells and CD8⁺ cytotoxic T cells, exerts pleiotropic effects in the tumour microenvironment, and its impact is dependent on the duration and magnitude of signalling 58. The immunosuppressive landscape of MI UC tissues, characterized by an ineffective T-cell response (Figure 3D, 3H), may enable prolonged IFN-γ exposure, thereby inducing MHC-II expression in cancer cells, as validated by *in vitro* IFN-γ treatment (Figure 6I-K, S7B-G).

Although MHC-II⁺ cancer cells constitute approximately 20% of MI basal tumours (Figure 6C and 6D), they may have a disproportionate functional effect on the tumour microenvironment. Increased expression of MHC-II molecules on cancer cells has also been observed in other advanced malignancies, including metastatic lymph nodes of breast cancer 56 and high-grade serous ovarian cancer 59. These findings suggest that a subcluster of cancer cells with elevated MHC-II expression may emerge under selective immune pressure, potentially contributing to immune modulation and tumour progression. In our study, MHC-II⁺ cancer cells were spatially colocalized with both CD8⁺ T cells and Tregs (Figure 8A-F), which indicates potential cellular interactions that may drive CD8⁺ T-cell exhaustion and facilitate immune evasion. In support of this, coculture experiments have shown that HLA-DR^hi^ cancer cells upregulate the inhibitory receptor expression on CD8⁺ T cells, including *PD-1*, *LAG-3*, and *TIM-3* 55. Additionally, MHC-II^+^ cancer cells have been reported to promote immune tolerance via Treg differentiation and expansion 56. Although the absence of costimulatory signals prevents the direct activation of T cells, our study revealed that MHC-II⁺ cancer cells exhibit upregulated *CD47* expression (Figure S10C), which enhances their interaction with SIRPα on macrophages (Figure 9C) and transmits a "don't eat me" signal that inhibits phagocytosis. By evading macrophage-mediated clearance, MHC-II⁺ cancer cells may facilitate immune escape; however, further research is needed to validate and clarify the mechanism.

In addition to their role in immune regulation, MHC-II⁺ cancer cells may also contribute to tumour progression through phenotypic changes. Overexpression of *HLA-DRA in vitro* enhanced cancer cell proliferation and migration (Figure 7G-K). The association between *HLA-DR* expression and cancer metastasis and aggressiveness was initially debated but were later substantiated. Recent studies have indicated that high MHC-II expression is correlated with functional stem cell activity in haematopoietic stem cells, and expression levels progressively decrease during differentiation 60. Moreover, MHC-II signalling, particularly via *HLA-DR*, has been implicated in promoting cancer cell migration and invasion by upregulating the expression of integrins and cell adhesion molecules and concurrently activating the JAK/STAT3 and PI3K/AKT pathways 61.

The tumour microenvironment undergoes dynamic evolution during cancer progression, and transitions from an immunostimulatory state to an immunosuppressive state. In our analysis, MI UC samples exhibited an immunosuppressive landscape characterized by a reduction in effector T cells, increased CD8^+^ T-cell exhaustion, increased Treg expansion, and a greater abundance of M2-polarized macrophages (Figure 3D, 4D), which may contribute to tumour progression and immune evasion. Exhausted CD8^+^ T cells lose their cytotoxic function, which enables unchecked tumor proliferation. The overexpression of inhibitory receptors on T cells, along with their ligands on cancer cells, facilitates immune evasion 45. Tregs suppress effector T-cell responses by secreting cytokines such as IL-10 and TGF-β and interacting by CD80/CD86 on DCs, thereby impairing costimulatory signalling 62. M2 macrophages, including well-characterized SPP1^+^ subsets, support tumor progression and metastasis through angiogenesis, extracellular matrix remodelling, and the secretion of immunosuppressive cytokines and chemokines 63, 64.

This study has several limitations. First, although our findings were derived from multiomics analyses and were validated in multiple independent cohorts, the experimental evidence remains relatively limited. In particular, the functional assays were primarily conducted *in vitro*, which may not fully recapitulate the complexity of the *in vivo* tumour microenvironment. Second, although our results suggest potential communication between MHC-II⁺ cancer cells and immune cell populations, the identified cellular interactions and regulatory pathways lack experimental validation. Additional *in vivo* studies and mechanistic experiments, such as those involving coculture systems and humanized mouse models, are needed to confirm and extend our observations.

In conclusion, our study provides a comprehensive single-cell analysis of UC, and reveals an immunosuppressive tumour microenvironment and a subset of cancer cells with MHC-II expression in MI tumours. The intricate cellular crosstalk between MHC-II⁺ cancer cells and immune cells facilitates immune evasion and tumour progression. However, further studies are needed to validate these findings and to elucidate their therapeutic implications.

## Supplementary Material

Supplementary figures and tables.

## Abbreviations

UC: urothelial carcinoma
MI: muscle-invasive
NMI: non-muscle-invasive
MHC-II: major histocompatibility complex II
UCB: urothelial carcinoma of the bladder
UTUC: upper tract urothelial carcinoma
scRNA-seq: single-cell RNA sequencing
NMF: nonnegative matrix factorization
cNMF: consensus nonnegative matrix factorization
MP: meta-program
IFN-γ: Interferon-γ
R_o/e_: **r**atio of **o**bserved to **e**xpected cell counts
ECs: endothelial cells
IUPU-UC: Institution of Urology, Peking University- urothelial carcinoma
TCGA-BLCA: The Cancer Genome Atlas-bladder cancer
CNV: copy number variation
IF: immunofluorescence
KL: Kullback-Leibler
