# Supplementary material for: Single-cell and spatial transcriptomic analysis reveal cellular heterogeneity and cancer cell-intrinsic major histocompatibility complex II expression in urothelial carcinoma
Source: Int J Biol Sci. 2025 Oct 20;21(15):6649–73. doi: 10.7150/ijbs.114618 (PMC12631105; doi:10.7150/ijbs.114618)
Supplement: Supplementary file 1 — Supplementary figures and tables. [file ijbsv21p6649s1.pdf]

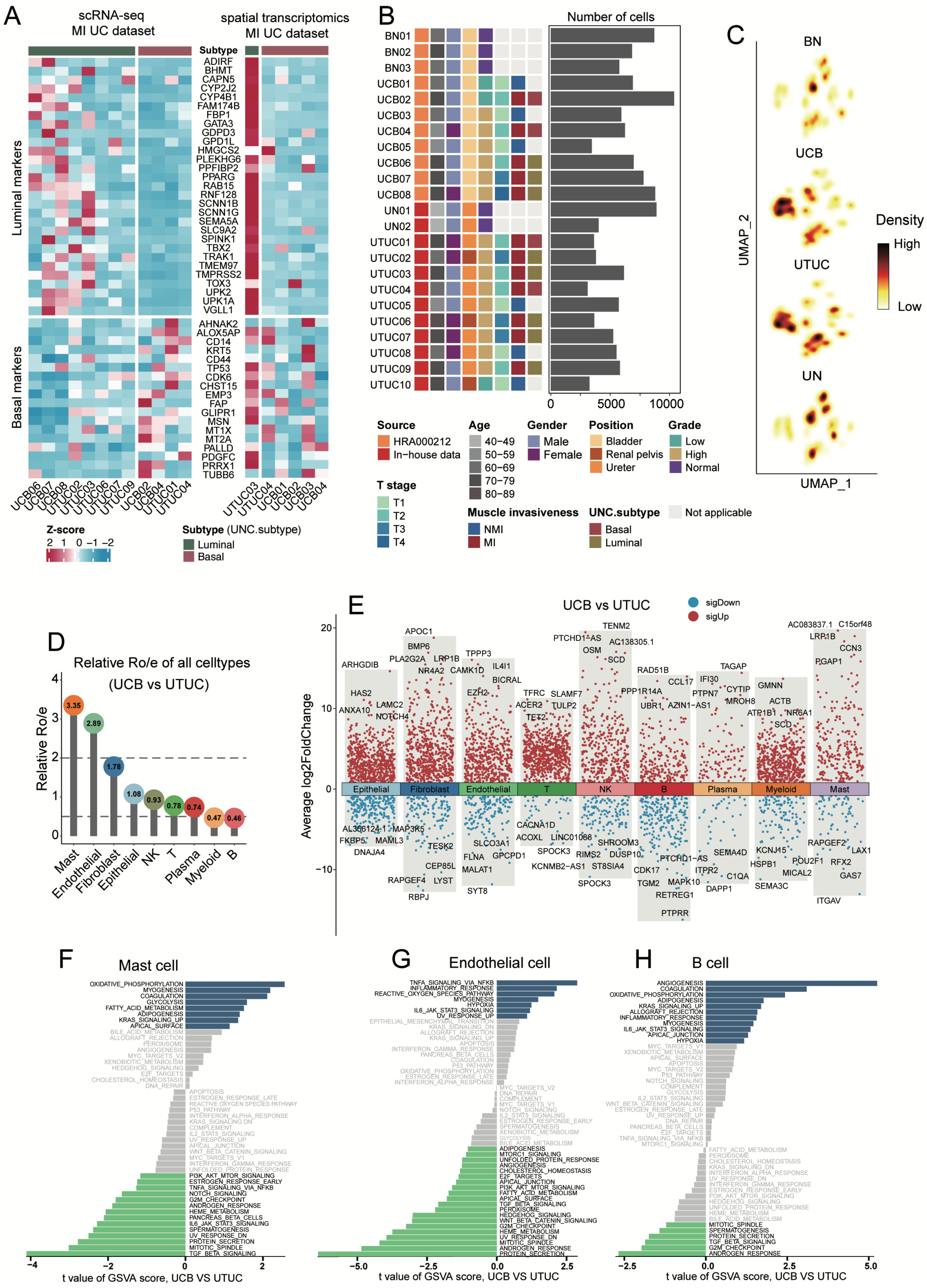

Figure S1 Distribution and heterogeneity of major cell types between UTUC and UCB, related to Figure 1. (A) Transcriptomic molecular subtypes of muscle-invasive (MI) urothelial carcinoma (UC) identified in scRNA-seq and spatial transcriptomics datasets. (B) Clinical and molecular characteristics of patients and cell counts per sample. (C) Cell density distributions across tissue subtypes. (D) Relative $R_{o/e}$ values of nine cell types compared between UCB and UTUC. (E) Volcano plot displaying the top five differentially expressed genes for each of the nine cell types in UCB compared to UTUC. (F-H) Gene set variation analysis (GSVA) of Hallmark pathway enrichment in (F) mast cells, (G) endothelial cells, and (H) B cells in UCB versus UTUC. Abbreviations: UTUC, upper tract urothelial carcinomas; UN, normal ureteral mucosa; UCB, urothelial carcinomas of the bladder; BN, normal bladder mucosa; NMI, non-muscle invasive; MI, muscle invasive.

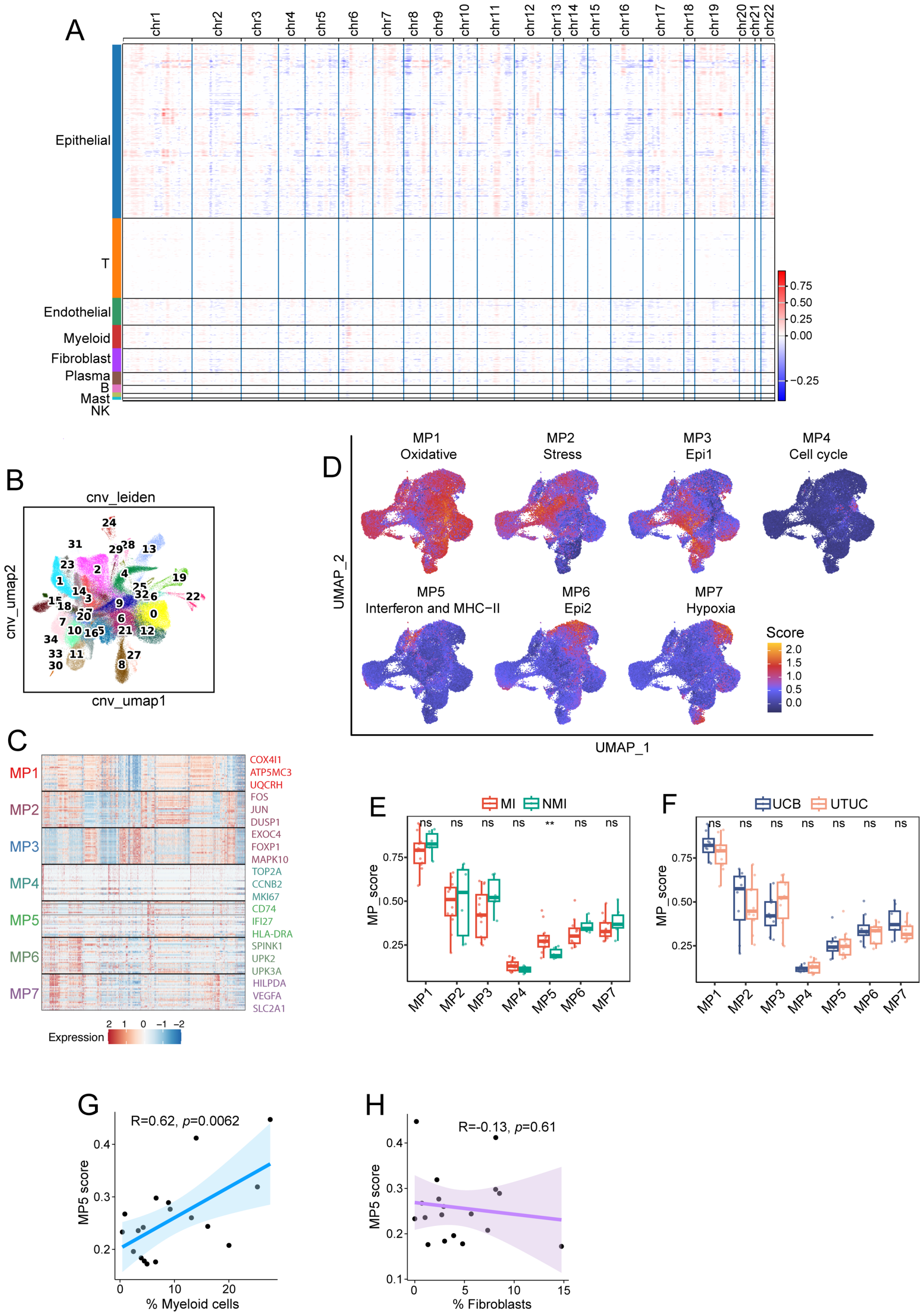

Figure S2 Analysis of intratumour heterogeneity of cancer cells in UC, related to Figure 2. (A) Heatmap displaying copy number variation (CNV) patterns grouped by cell types across 18 UC samples. (B) UMAP plot of CNV clusters. Epithelial cells with high CNV scores were classified as cancer cells, while clusters with low CNV scores resembling immune cells were regarded as normal epithelial cells. (C) Heatmap of scaled expression for three representative genes from each meta-program (MP). (D) UMAP plots of cancer cells, colored by the normalized signature scores for each MP. MP names reflect their most enriched pathways. (E) Comparison of MP scores between muscle invasive (MI) and non-muscle invasive (NMI) groups. (G, H) Spearman correlation analysis between MP5 score and cell proportion of myeloid cells(G) and fibroblasts (H) in UC tumor samples.

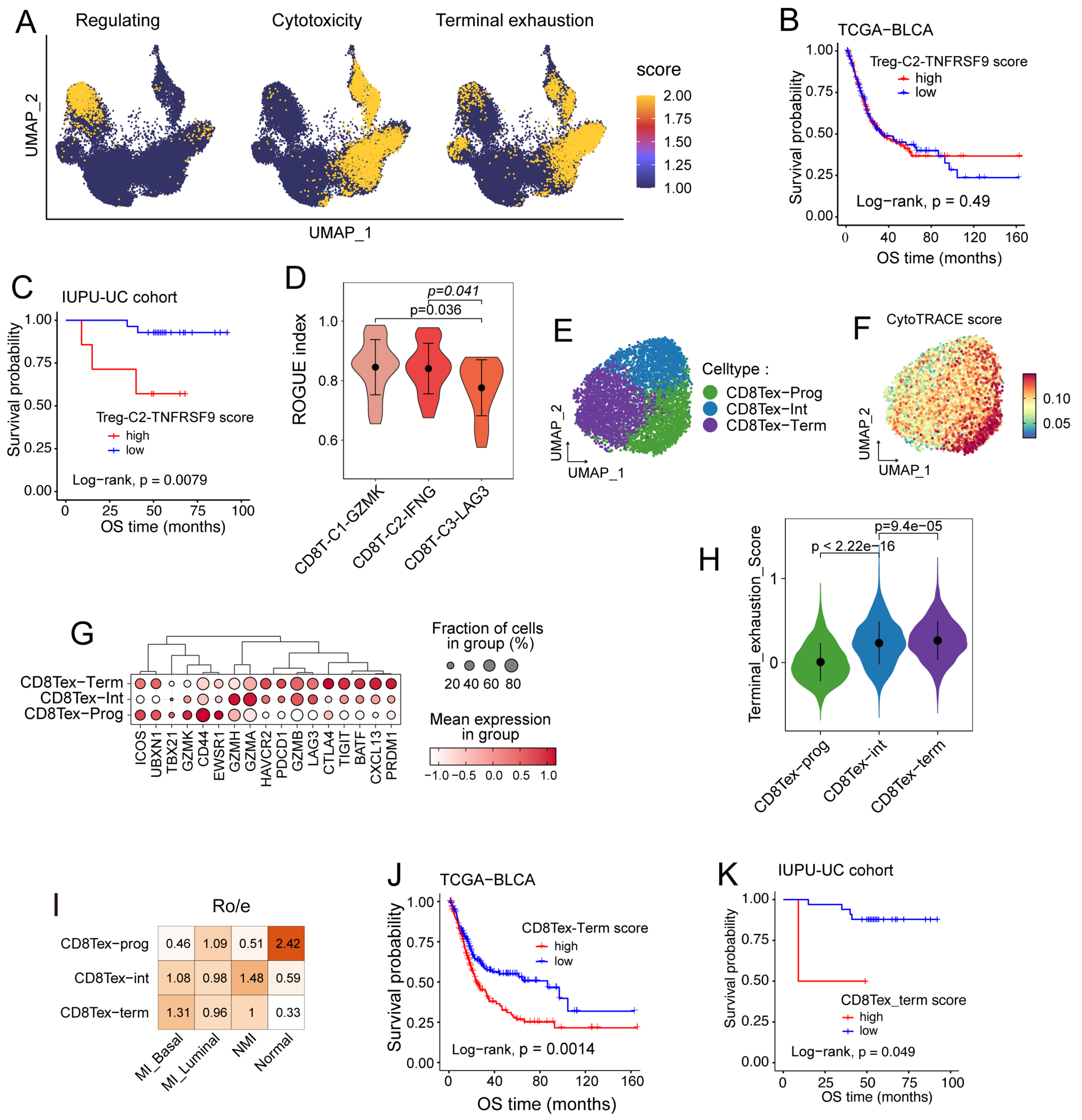

Figure S3 Characteristics of T and NK cells, related to Figure 2. (A) UMAP plots showing regulating, cytotoxicity, and terminal exhaustion signature scores of T and NK cell clusters. (B, C) Kaplan-Meier survival analysis of overall survival (OS) in the (B) TCGA-BLCA and (C) IUPU-UC cohorts, stratified by high versus low Treg-C2-TNFRSF9 signature scores. (D) Violin plot showing cell purity among the three $CD8^{+}$ T cell subsets, as assessed by ROGUE analysis. (E, F) UMAP plots of exhausted $CD8^{+}$ T cells (CD8Tex), colored by cell subsets (E) and CytoTRACE scores (F). (G) Dot plot showing expression of key marker genes in the three CD8Tex subsets. (H) Violin plots of terminal exhaustion scores in the three $CD8^{+}$ T cell subsets. (I) $R_{o/e}$ values indicating pathologic and molecular subtype preferences among CD8Tex subsets. (J, K) Kaplan-Meier analysis of overall survival in the TCGA-BLCA (J) and in-house IUPU-UC (K) cohorts, stratified by high versus low CD8Tex-Term signature scores.

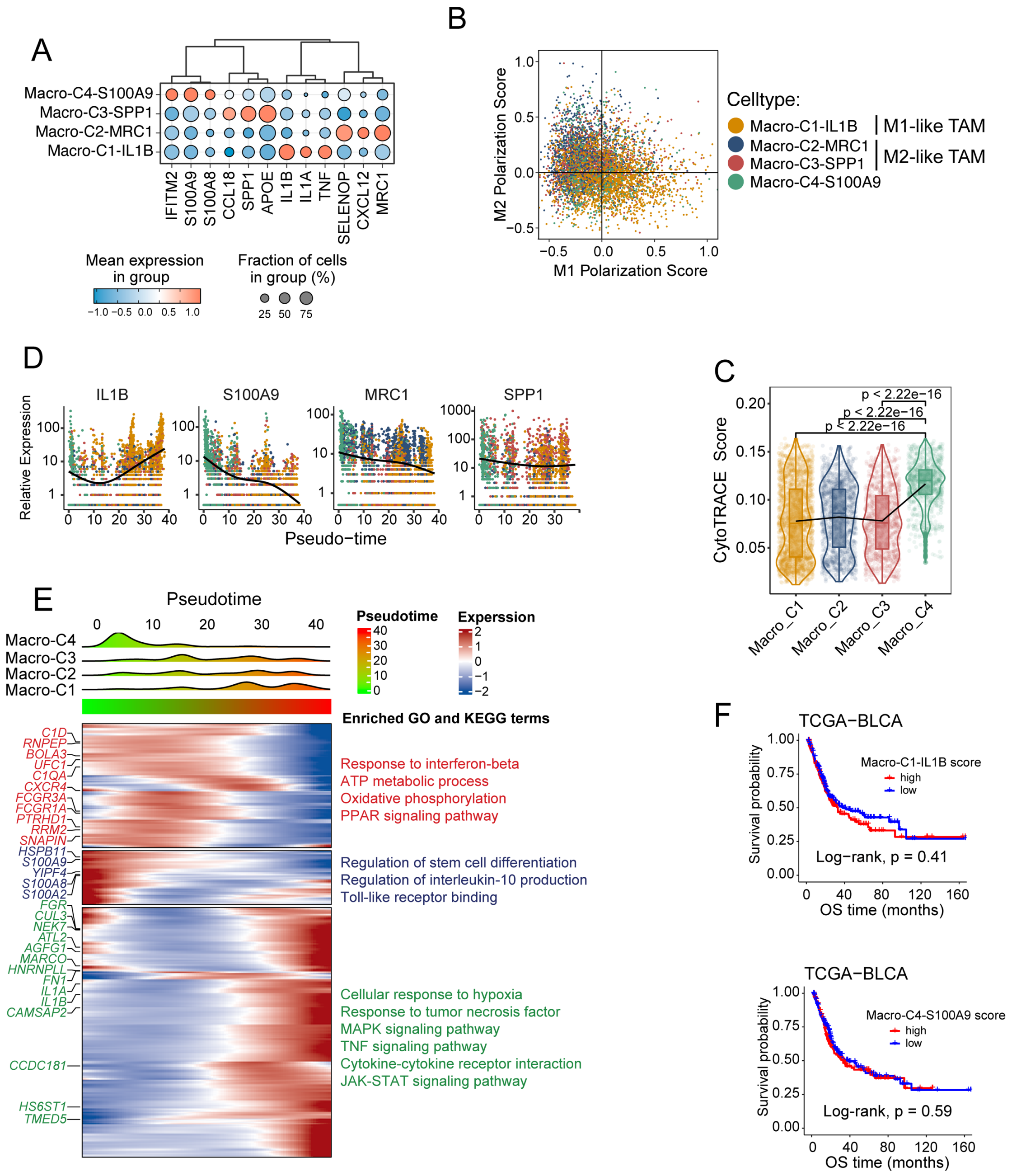

Figure S4 Characteristics of macrophages subclusters, related to Figure 4. (A) Bubble plot showing marker genes across four macrophage subsets. (B) scatter plot showing the M1 and M2 polarization scores across four macrophage clusters. (C) Violin plot showing the CytoTRACE scores across four macrophage clusters. (D) Scatter plot illustrating the marker gene expression along the pseudotime across the four macrophage subsets. (E) Heatmap of dynamic gene expression changes across macrophage subsets. Normalized expression and pseudotime are represented by color gradients (top). Genes are hierarchically clustered (left), with enriched GO terms and KEGG pathways shown (right). (F) Kaplan–Meier analysis of overall survival (OS) in the TCGA-BLCA cohort stratified by high versus low infiltration of macro-C1-IL1B (top) and macro-C4-S100A9 (bottom). Significance was assessed using the log-rank test.

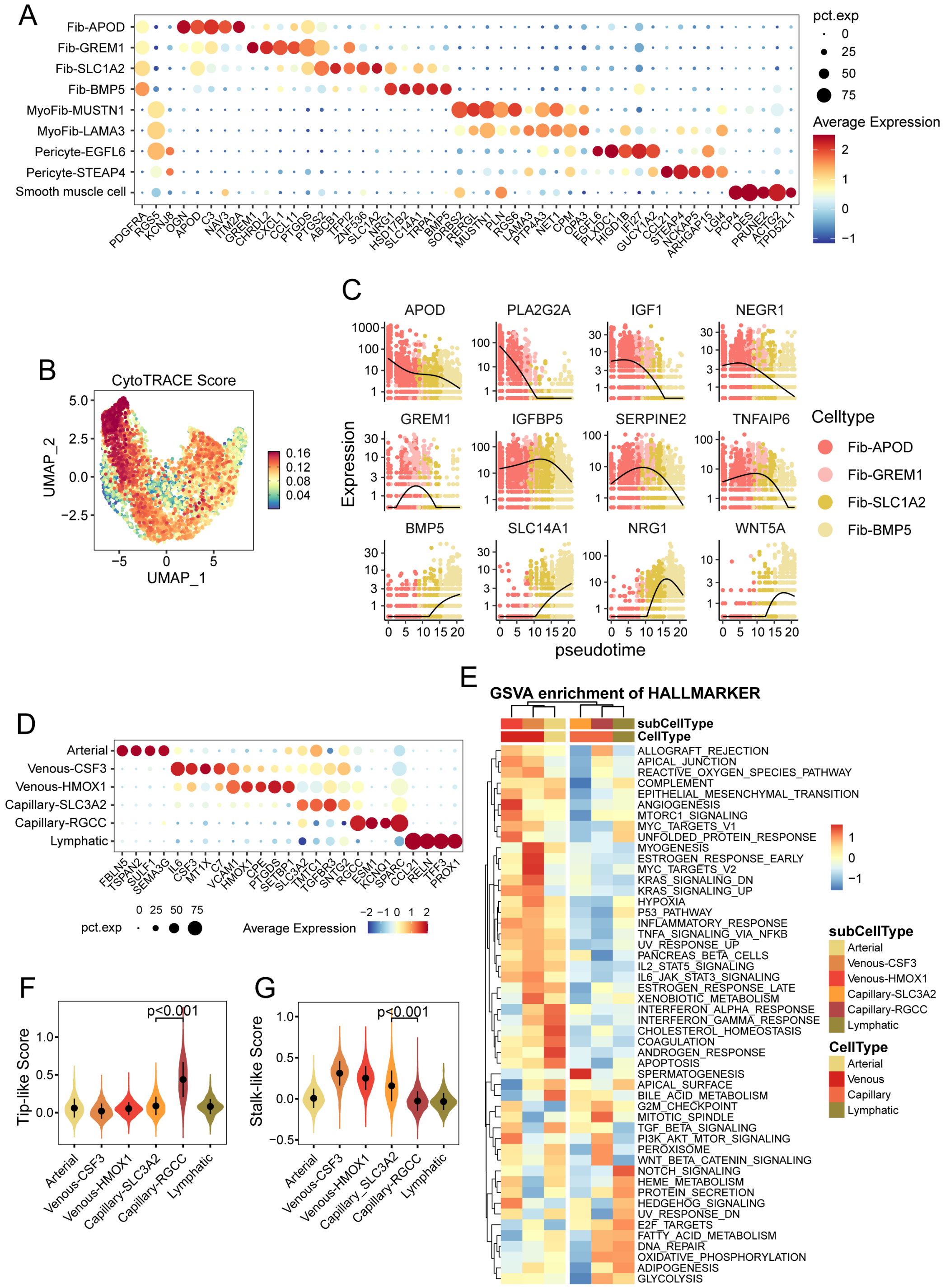

Figure S5 Characteristics of fibroblast and endothelial cell subclusters, related to Figure 5. (A) Bubble plot showing differentially expressed genes across nine stromal cell subsets. (B) UMAP visualization of CytoTRACE scores across four fibroblast subclusters. (C) Scatter plot illustrating dynamic changes in key marker gene expression along pseudotime trajectories in four fibroblast subsets. (D) Bubble plot showing differentially expressed genes across six endothelial cell subsets. (E) Heatmap of GSVA enrichment scores for hallmark pathways across endothelial cell subclusters. (F, G) Violin plots showing the distribution of tip-like (F) and stalk-like (G) signature scores across endothelial cell subclusters.

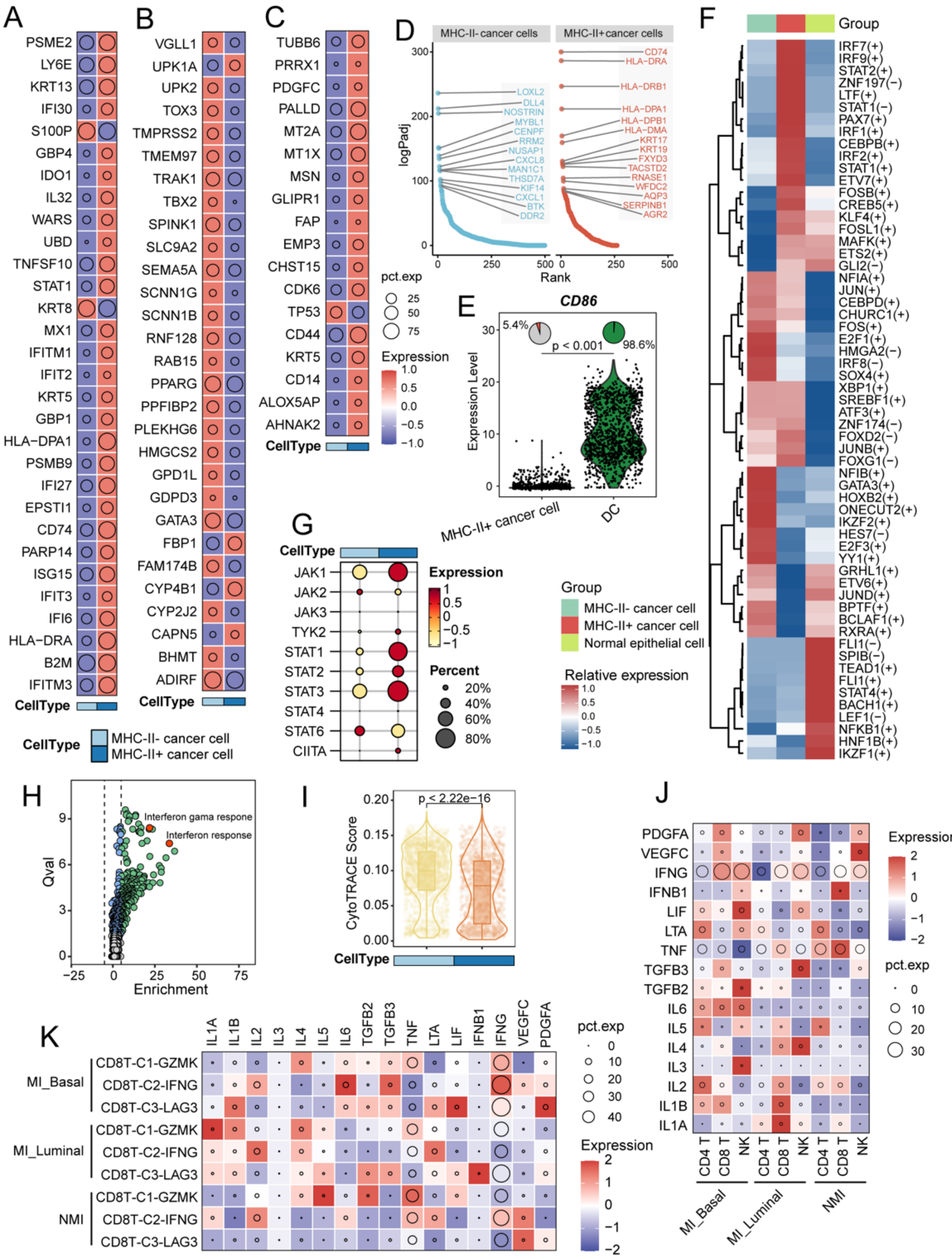

Figure S6 Identification of MHC-II^{+} cancer cells in UC, related to Figure 6. (A-C) Bubble plot showing the normalized expression of MP5 signatures (A), luminal phenotype signatures (B), and basal phenotype signatures (C) across cancer cell subsets. (D) Gene ranking dot plot of differentially expressed genes between MHC-II^{+} and MHC-II^{-} cancer cells. (E) Violin plot comparing *CD86* expression between MHC-II^{+} cancer cells versus dendritic cells (DCs). (F) Heatmap showing transcription factor expression in MHC-II^{+} cancer cells, MHC-II$^{–}$ cancer cells, and normal epithelial cells. (G) Bubble plot showing expression of JAK/STAT pathway genes in MHC-II^{+} and MHC-II$^{–}$ cancer cells. (H) Volcano plot of enriched pathways in MHC-II+ cancer cells. (I) Violin plot of CytoTRACE scores across cancer cell subsets. (J, K) Bubble plots displaying normalized expression of cytokine genes across T cell subsets and NK cells (J), and CD8^{+} T cell subsets (K).

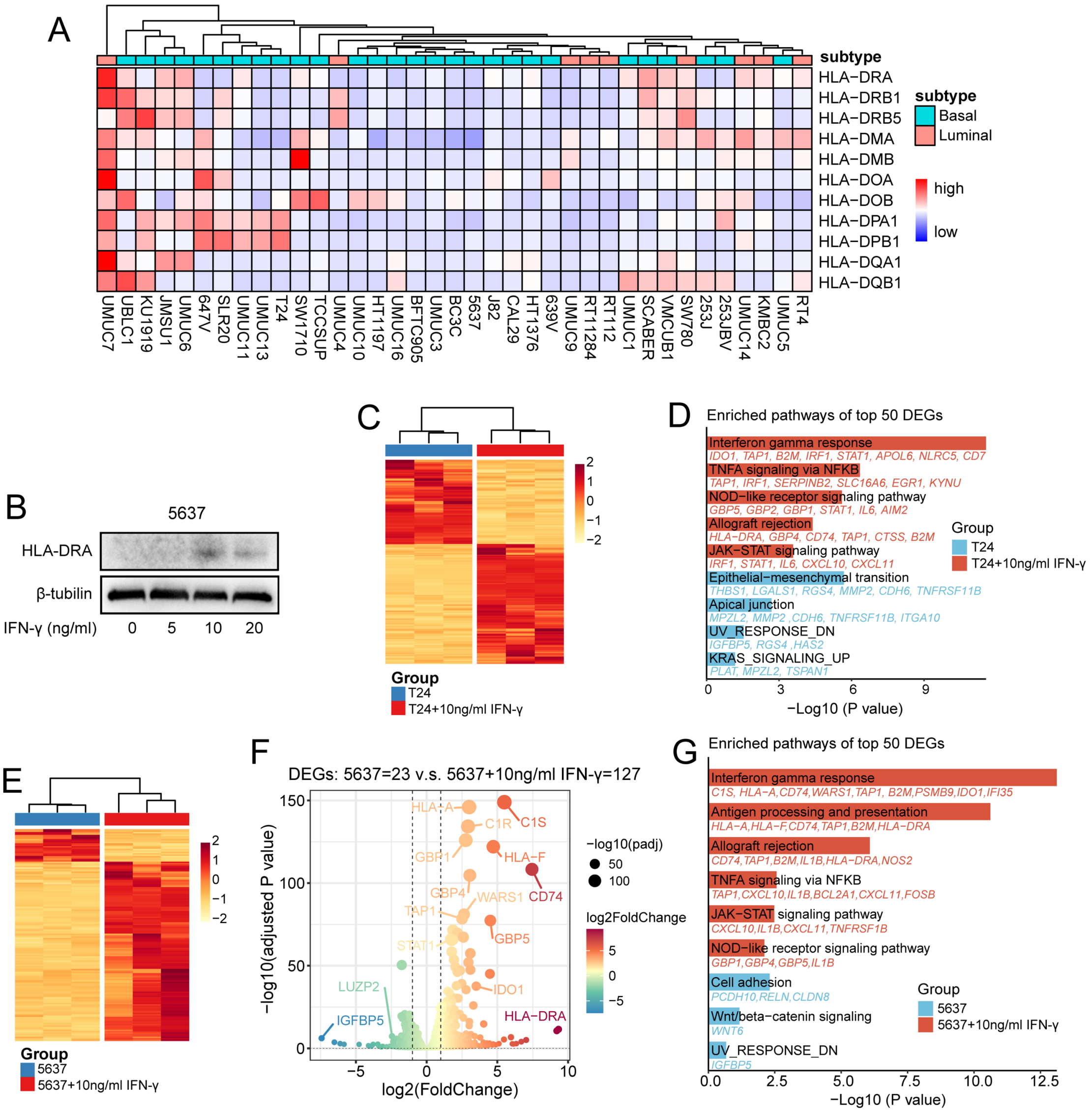

Figure S7 IFN-γ signalling drives the expression of MHC-II molecules on cancer cells, related to Figure 6. (A) Heatmap of normalized MHC-II molecule expression across 37 bladder cancer cell lines from the Cancer Cell Line Encyclopedia (CCLE) database, stratified by luminal and basal subtypes. (B) Western blot showing IFN-γ–induced upregulation of HLA-DRA expression in 5637 cells treated with IFN-γ (0, 5, 10, 20 ng/mL) for 72 hours. (C, D) Clustering analysis (C) and functional enrichment (D) of differentially expressed genes (DEGs) between T24 cells treated with or without 10 ng/mL IFN-γ. Color represents normalized expression values. (E–G) Clustering analysis (E), volcano plot (F), and functional enrichment (G) of DEGs between 5637 cells treated with or without 10 ng/mL IFN-γ.

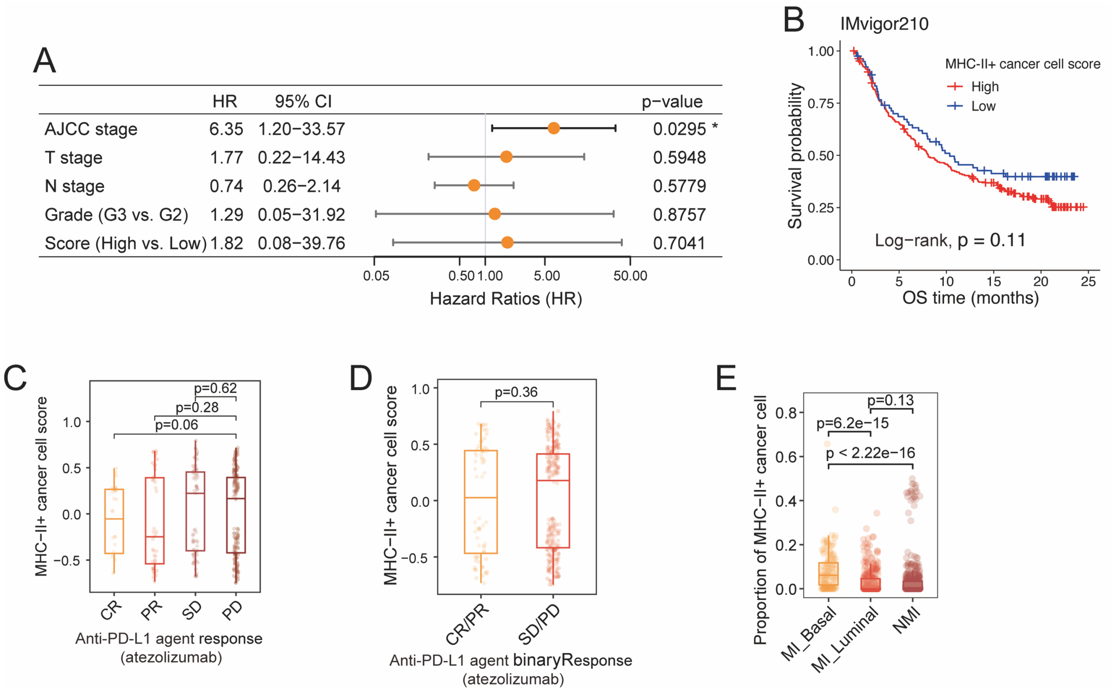

Figure S8 MHC-II^{+} cancer cells predict poor prognosis, related to Figure 7. (A) Multivariate Cox regression analysis of the MHC-II^{+} cancer cell signature score and clinicopathological factors in the IUPU-UC cohort. (B) Kaplan–Meier analysis of overall survival (OS) in the IMvigor210 cohort stratified by high versus low MHC-II^{+} cancer cell signature scores. (C, D) Box plots showing MHC-II^{+} cancer cell signature scores by detailed response type (C) and binary response(D) to the anti-PD-L1 agent atezolizumab in the IMvigor210 cohort. (E) Comparison of estimated proportions of MHC-II^{+} cancer cells across molecular subgroups. Colored dots represent individual samples. Abbreviations: AJCC, American Joint Committee on Cancer; CR, complete response; PR, partial response; SD, stable disease; PD, progressive disease.

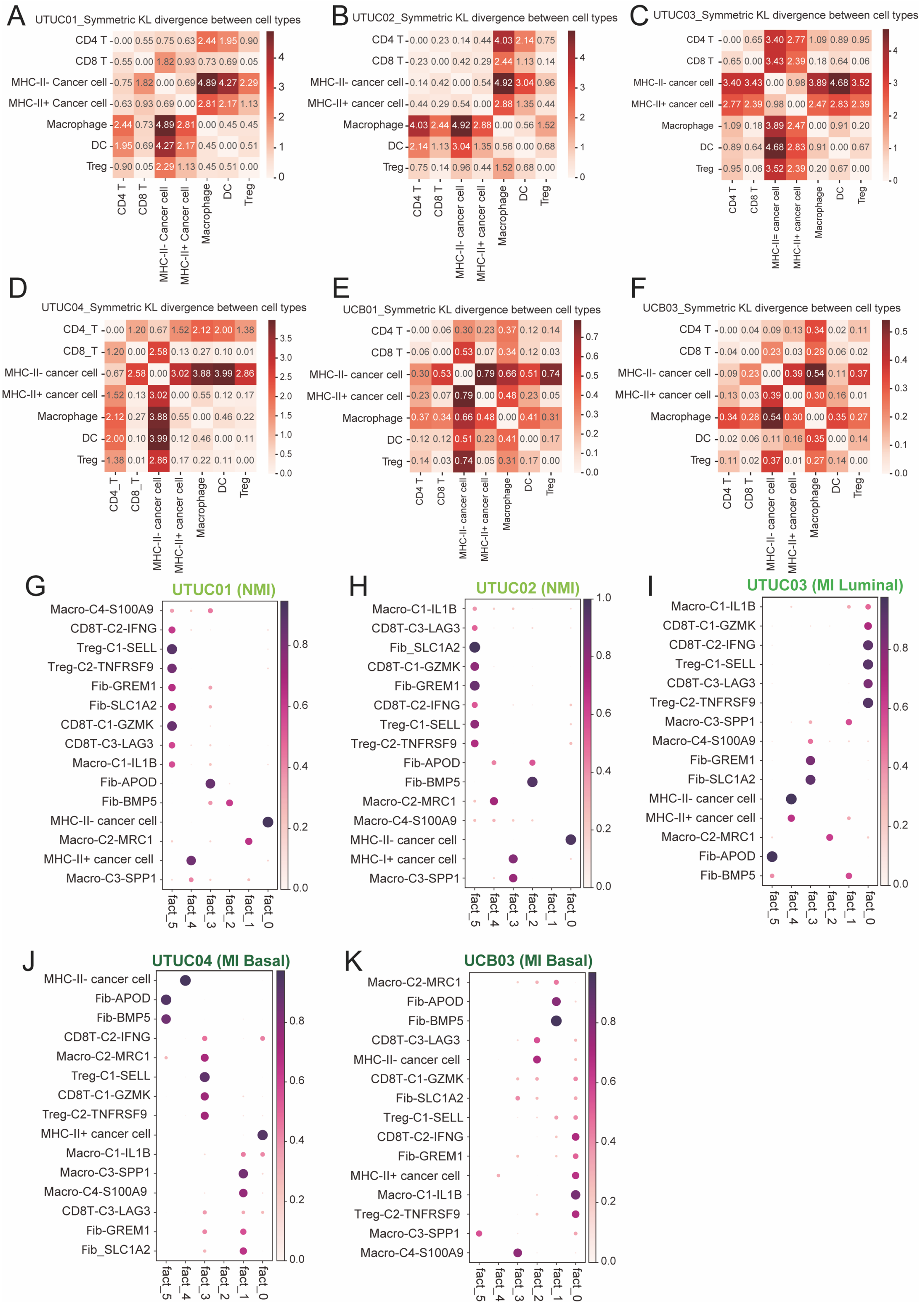

Figure S9 Spatial colocalization of MHC-II^{+} cancer cell and immunecell subclusters, related to Figure 8. (A-F) Heatmaps of the Kullback-Leibler (KL) divergence between major cell subsets, providing a global view of spatial similarity among all cell types. (G-K) Identification of cell compartments using non-negative matrix factorization (NMF) in UTUC and UCB tumor sections, showing normalized weights of each cell type across NMF components. Color intensity represents weight values.

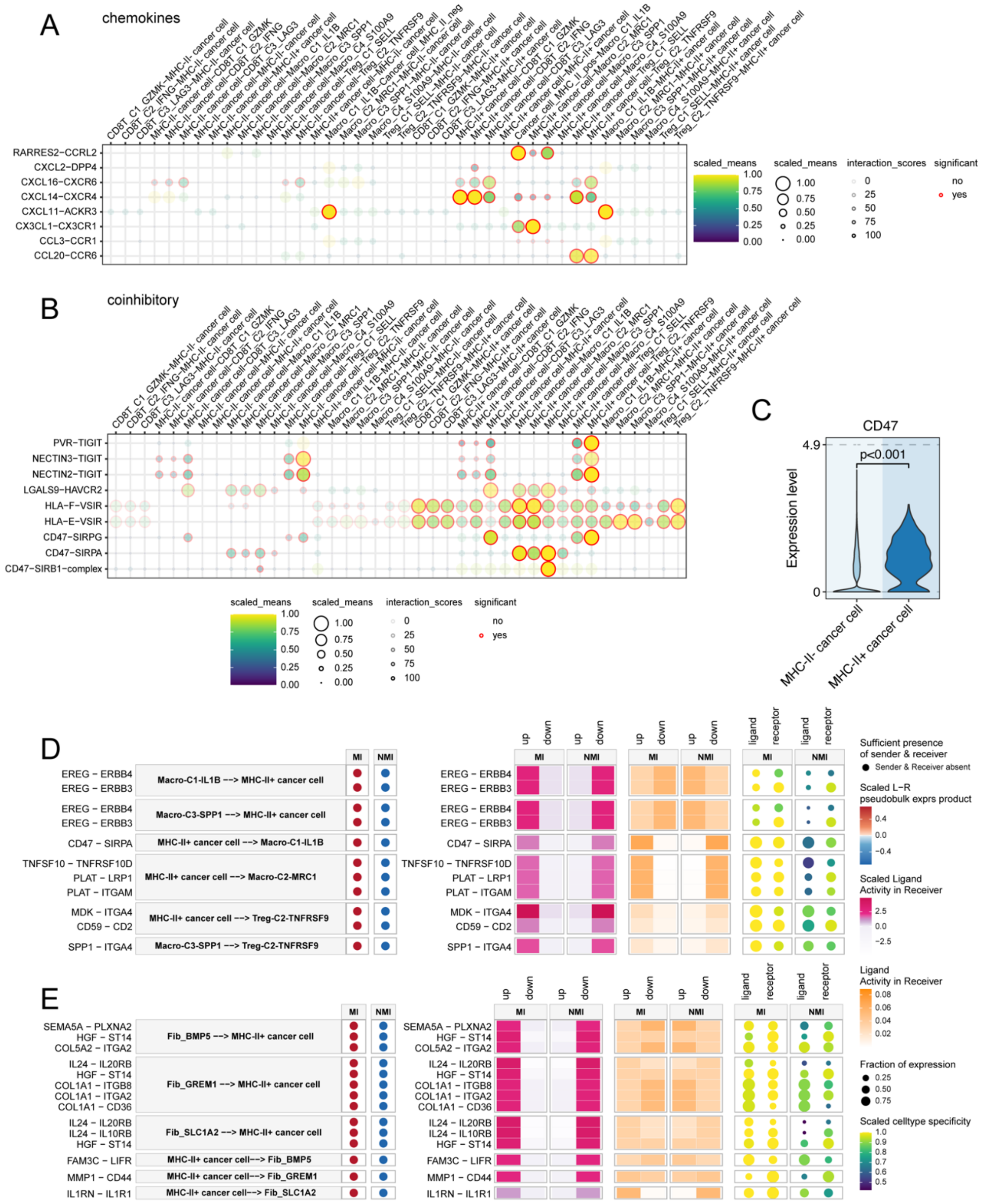

Figure S10 Interaction networks among cell subclusters, related to Figure 9. (A, B) Bubble plots showing ligand–receptor interactions involved in chemokine signalling (A) and costimulatory/coinhibitory molecules (B) across cell subclusters. (C) Violin plot showing expression differences of *CD47* among cancer cell subsets in UC. (D, E) MultiNicheNet analysis of top ligand–receptor interactions between MHC-II^{+} cancer cells and immune cells (D) or fibroblasts (E) in muscle-invasive (MI) versus non-muscle-invasive (NMI) UC samples.

Table S1 Clinicopathological and molecular characteristics of the UC patients and normal donors profiled by single-cell RNA sequencing.

Sample ID	Data source	Gender	Age, years	Tumor or tissue location	Tumor T stage	Tumor muscle invasiveness	Tumor grade	Molecular subtype	Cell number after QC
UCB01	HRA000212	Male	67	Bladder	T1	Non-muscle invasive	Low	-	6905
UCB02	HRA000212	Male	70	Bladder	T2	Muscle invasive	Low	Basal	10386
UCB03	HRA000212	Male	63	Bladder	T1	Non-muscle invasive	High	-	5943
UCB04	HRA000212	Female	59	Bladder	T2	Muscle invasive	High	Basal	6246
UCB05	HRA000212	Male	57	Bladder	T1	Non-muscle invasive	High	-	3449
UCB06	HRA000212	Male	75	Bladder	T2	Muscle invasive	High	Luminal	6980
UCB07	HRA000212	Male	77	Bladder	T3	Muscle invasive	High	Luminal	7802
UCB08	HRA000212	Female	72	Bladder	T4	Muscle invasive	High	Luminal	8786
UTUC01	In-house data, HRA001867	Female	74	Ureter	T2	Muscle invasive	High	Basal	3642
UTUC02	In-house data, HRA001867	Female	69	Renal pelvis	T3	Muscle invasive	High	Luminal	3802
UTUC03	In-house data, HRA001867	Male	72	Ureter	T2	Muscle invasive	High	Luminal	6164
UTUC04	In-house data, HRA001867	Male	61	Ureter	T2	Muscle invasive	High	Basal	3098
UTUC05	In-house data, HRA001867	Male	58	Ureter	T1	Non-muscle invasive	High	-	5723
UTUC06	In-house data, HRA001867	Female	81	Renal pelvis	T3	Muscle invasive	High	Luminal	3660

UTUC07	In-house data, HRA001867	Female	70	Ureter	T3	Muscle invasive	High	Luminal	5252
UTUC8	In-house data, HRA001867	Female	65	Ureter	T1	Non-muscle invasive	High	-	5530
UTUC9	In-house data, HRA001867	Male	78	Ureter	T2	Muscle invasive	High	Luminal	5815
UTUC10	In-house data, HRA001867	Male	77	Renal pelvis	T1	Non-muscle invasive	Low	-	3260
BN01	HRA000212	Male	67	Bladder					8711
BN02	HRA000212	Male	75	Bladder					6844
BN03	HRA000212	Male	63	Bladder					5771
UN01	In-house data	Male	40	Ureter					8891
UN02	In-house data	Male	45	Ureter					4027

UCB, urothelial carcinoma of bladder; UTUC, upper tract urothelial carcinoma; UN, ureter normal urothelium; BN: bladder normal urothelium;

Table S2 Clinicopathologic and molecular characteristics of UC patients profiled by spatial transcriptomics.

Sample ID	Data source	Gender	Age, years	Tumor location	Tumor T stage	Tumor muscle invasiveness	Tumor grade	Molecular subtype	Spot number
UCB01	GSE171351	-	-	Bladder	-	Muscle invasive	-	Basal	777
UCB02	GSE171351	-	-	Bladder	-	Muscle invasive	-	Basal	970
UCB03	GSE171351	-	-	Bladder	-	Muscle invasive	-	Basal	1024
UCB04	GSE171351	-	-	Bladder	-	Muscle invasive	-	Basal	1584
UTUC01	In-house data	Female	54	Ureter	T1	Non-muscle invasive	High	-	2031
UTUC02	In-house data	Male	60	Ureter	T1	Non-muscle invasive	High	-	3266
UTUC03	In-house data	Female	72	Renal pelvis	T3	Muscle invasive	High	Luminal	3283
UTUC04	In-house data	Female	77	Ureter	T3	Muscle invasive	High	Basal	2283

UCB, urothelial carcinoma of bladder; UTUC, upper tract urothelial carcinoma

Table S3 Clinicopathological characteristics of patients from the in-house IUPU-UC cohort profiled by bulk RNA sequencing.

Sample ID	Data source	Gender	Age, years	Tumor or tissue location	Tumor T stage	Tumor muscle invasiveness	Tumor grade
UTUC01	in-house data	61	Male	Pelvis	T1	Non-muscle invasive	High
UTUC02	in-house data	55	Male	Pelvis	T1	Non-muscle invasive	High
UTUC03	in-house data	48	Male	Pelvis	T1	Non-muscle invasive	High
UTUC04	in-house data	72	Male	Ureter	T1	Non-muscle invasive	Low
UTUC05	in-house data	75	Female	Pelvis	T1	Non-muscle invasive	High
UTUC06	in-house data	63	Male	Ureter	T1	Non-muscle invasive	High
UTUC07	in-house data	53	Female	Pelvis	T1	Non-muscle invasive	Low
UTUC08	in-house data	71	Female	Pelvis	T1	Non-muscle invasive	Low
UTUC09	in-house data	57	Male	Pelvis	T1	Non-muscle invasive	High
UTUC10	in-house data	68	Female	Pelvis	T1	Non-muscle invasive	High
UTUC11	in-house data	65	Male	Pelvis	T1	Non-muscle invasive	Low
UTUC12	in-house data	69	Male	Pelvis	T1	Non-muscle invasive	Low
UTUC13	in-house data	70	Male	Pelvis	T1	Non-muscle invasive	Low
UTUC14	in-house data	41	Male	Ureter	T1	Non-muscle invasive	Low
UTUC15	in-house data	76	Female	Ureter	T1	Non-muscle invasive	High
UTUC16	in-house data	63	Female	Pelvis	T1	Non-muscle invasive	High

UTUC17	in-house data	85	Male	Ureter	T2	Muscle invasive	High
UTUC18	in-house data	78	Female	Pelvis	T3	Muscle invasive	High
UTUC19	in-house data	50	Female	Pelvis	T3	Muscle invasive	High
UTUC20	in-house data	77	Female	Pelvis	T4	Muscle invasive	High
UTUC21	in-house data	63	Female	Ureter	T3	Muscle invasive	High
UTUC22	in-house data	60	Male	Ureter	T2	Muscle invasive	High
UTUC23	in-house data	48	Female	Pelvis	T4	Muscle invasive	High
UTUC24	in-house data	80	Female	Pelvis	T3	Muscle invasive	High
UTUC25	in-house data	49	Male	Pelvis	T2	Muscle invasive	Low
UTUC26	in-house data	78	Male	Pelvis	T3	Muscle invasive	High
UTUC27	in-house data	64	Male	Ureter	T3	Muscle invasive	High
UTUC28	in-house data	83	Female	Pelvis	T3	Muscle invasive	High
UTUC29	in-house data	67	Male	Pelvis	T2	Muscle invasive	Low
UCB01	in-house data	73	Male	Bladder	T2	Muscle invasive	High
BN01	in-house data	73	Male	Bladder	Normal		
UCB02	in-house data	67	Male	Bladder	T3	Muscle invasive	High
BN02	in-house data	67	Male	Bladder	Normal		
UCB03	in-house data	76	Male	Bladder	T1	Non-muscle invasive	Low

BN03	in-house data	76	Male	Bladder	Normal		
UCB04	in-house data	42	Male	Bladder	T1	Non-muscle invasive	High
BN04	in-house data	42	Male	Bladder	Normal		
UCB05	in-house data	83	Female	Bladder	T1	Non-muscle invasive	Low
BN05	in-house data	83	Female	Bladder	Normal		
UCB06	in-house data	54	Male	Bladder	T1	Non-muscle invasive	Low
BN06	in-house data	54	Male	Bladder	Normal		

Table S4 Functionally annotated gene sets used in this study

Gene sets	Gene symbols
Luminal phenotype	*ADIRF, BHMT, CAPN5, CYP2J2, CYP4B1, FAM174B, FBP1, GATA3, GDPD3, GPD1L, HMGCS2, PLEKHG6, PPFIBP2, PPARG, RAB15, RNF128, SCNN1B, SCNN1G, SEMA5A, SLC9A2, SPINK1, TBX2, TRAK1, TMEM97, TMPRSS2, TOX3, UPK2, UPK1A, VGLL1*
Basal phenotype	*AHNAK2, ALOX5AP, CD14, KRT5, CD44, TP53, CDK6, CHST15, EMP3, FAP, GLIPR1, MSN, MT1X, MT2A, PALLD, PDGFC, PRRX1, TUBB6*
Regulating	TNFRSF9, TNFRSF18, ENTPD1, IKZF4, LRRC32, IKZF2, STAT5A, FOXP3, CD4
Cytotoxicity	GZMB, GZMH, GZMK, GZMA, TIA1, PRF1, LAMP1, GNLY, FASLG, SLAMF7, ZAP70, CD69, TNF
Terminal exhaustion	*CXCL13, ENTPD1, PRF1, GZMH, GZMB, GZMA, BATF, LYST, BATF, TIGIT, LAG3, HAVCR2, PDCD1, PRDM1, RBPJ, SLAMF7, CCL4, TNFSF10, TBX21, NFATC1, HIF1A, TNFRSF4, CXCR6, PTPN7, EWSR1, HLA-DPB1, HLA-DPA1, UBXN1, PSMB9, LY6E, CCNDBP1, IRF9, LCP2*
Proliferation	*MKI67, IGF1, ITGB2, PDGFC, JAG1, PHGDH*
Migration	*VIM, SNAI1, MMP9, AREG, ARID5B, FAT1*
M1 polarization	*CXCL9, CXCL10, CXCL11, IRF1, IL1B, CD86, MARCO, IL12A, TNF, FCGR1A*
M2 polarization	*CLEC7A, GAS7, CCL18, CD209, LIPA, F13A1, CTSD, MS4A4A, MAF, CSF1R, CCL23, CCL7, CCL2, CCL17, HMOX1, FN1, IL27RA, CXCR4, PPARG*
Tip cells	*ADM, ANGPT2, ANKRD37, APLN, C1QTNF6, CD93, CLDN5, COL4A1, COL4A2, COTL1, CXCR4, DLL4, EDNRB, ESM1, FSCN1, GPIHBP1, HSPG2, IGFBP3, INHBB, ITGA5, JUP, KCNE3, KCNJ8, KDR, LAMA4, LAMB1, LAMC1, LXN, MARCKS, MARCKSL1, MCAM, MEST, MYH9, MYO1B, N4BP3, NID2, NOTCH4, PDGFB, PGF, PLOD1, PLXND1, PMEPA1, PTN, RAMP3, RBP1, RGCC, RHOC, SMAD1, SOX17, SOX4, SPARC, TCF4, UNC5B, VIM*
Stalk cells	*ACKR1, AQP1, C1QTNF9, CD36, CSRP2, EHD4, FBLN5, HSPB1, LIGP1, IL6ST, JAM2, LGALS3, LRG1, MEOX2, PLSCR2, SDPR, SELP, SPINT2, TGFBI, TGM2, TMEM176A, TMEM176B, TMEM252, TSPAN7, VEGFR1, VWF*

Table S5 Gene signature sets for seven meta-programs (MPs) of cancer cells in UC.

MP1	MP2	MP3	MP4	MP5	MP6	MP7
GSTP1	*ATF3*	*CCSER1*	*HMGB2*	*IFITM3*	*ADIRF*	*HILPDA*
NME2	*JUN*	*MECOM*	*BIRC5*	*B2M*	*PSCA*	*P4HA1*
UQCRH	*FOS*	*PARD3*	*CCNB1*	*HLA-DRA*	*SNCG*	*BNIP3*
H3F3A	*BTG2*	*LPP*	*PTTG1*	*IFI6*	*UPK1A*	*ENO1*
NACA	*PPP1R15A*	*PLCB1*	*TOP2A*	*IFIT3*	*GDPD3*	*ERO1A*
PPIA	*DUSP1*	*RERE*	*CCNB2*	*ISG15*	*SPINK1*	*FAM162A*
CHCHD2	*EGR1*	*IMMP2L*	*CDC20*	*PARP14*	*UPK1B*	*FTH1*
EEF1A1	*FOSB*	*LRBA*	*CENPF*	*CD74*	*UPK2*	*GAPDH*
RACK1	*IER2*	*MAPK10*	*MKI67*	*EPSTI1*	*UPK3A*	*LDHA*
EEF1G	*JUNB*	*NAALADL2*	*TPX2*	*IFI27*	*CLIC3*	*SLC2A1*
GAPDH	*SQSTM1*	*PATJ*	*UBE2C*	*PSMB9*	*PRR15L*	*CST6*
PFN1	*ZFP36*	*PTK2*	*NUSAP1*	*HLA-DPA1*	*S100P*	*EGLN3*
UBA52	*UBC*	*ZBTB20*	*TROAP*	*GBP1*	*VAMP8*	*EIF1*
BTF3	*DNAJB1*	*ARID1B*	*TUBA1B*	*KRT5*	*ERP27*	*ENO2*
COX4I1	*FOSL1*	*BCAS3*	*AURKB*	*IFIT2*	*CD24*	*FHL2*
FAU	*HSPA1A*	*EFNA5*	*CDK1*	*IFITM1*	*ANXA9*	*MIF*
LDHB	*HSPA1B*	*EXOC6B*	*CDKN3*	*MX1*	*VAMP5*	*NDRG1*
MYL12B	*DUSP2*	*PLEKHA5*	*CENPE*	*KRT8*	*RARRES1*	*PGK1*
PTMA	*HES1*	*RABGAP1L*	*DLGAP5*	*STAT1*	*HOPX*	*SLC6A8*
S100A11	*IER3*	*RAD51B*	*H2AFZ*	*TNFSF10*	*MAL*	*VEGFA*
SLC25A5	*KLF6*	*SDK1*	*HMGB1*	*UBD*	*HPGD*	*ADM*
TPT1	*NR4A1*	*TANC2*	*KIF4A*	*WARS*	*CAPG*	*BHLHE40*
ATP5MC2	*DNAJA1*	*DGKH*	*NUF2*	*IL32*	*RALBP1*	*BNIP3L*
TXN	*KLF4*	*EXOC4*	*STMN1*	*IDO1*	*UQCRQ*	*CSTB*
EEF1B2	*KRT17*	*FOXP1*	*ARL6IP1*	*GBP4*	*TMEM97*	*INSIG2*
S100A6	*MAFF*	*NF1*	*CDCA3*	*S100P*	*SPINT1*	*PPDPF*
ATP5MC3	*ZSWIM6*	*PBX1*	*ASPM*	*IFI30*	*MUC20*	*CITED2*
KRT7	*ZFAND2A*	*PTPRM*	*PBK*	*KRT13*	*NDRG2*	*DDIT3*
PRDX1	*BAG3*	*ZFAND3*	*SGO1*	*LY6E*	*TACSTD2*	*PDK1*
SERF2	*HSPA6*	*MSI2*	*CENPA*	*PSME2*	*SERF2*	*PGF*
